# Capturing the ‘ome’: the expanding molecular toolbox for RNA and DNA library construction

**DOI:** 10.1093/nar/gky167

**Published:** 2018-03-05

**Authors:** Morgane Boone, Andries De Koker, Nico Callewaert

**Affiliations:** 1 Center for Medical Biotechnology, VIB, Zwijnaarde 9052, Belgium; 2 Department of Biochemistry and Microbiology, Ghent University, Ghent 9000, Belgium

## Abstract

All sequencing experiments and most functional genomics screens rely on the generation of libraries to comprehensively capture pools of targeted sequences. In the past decade especially, driven by the progress in the field of massively parallel sequencing, numerous studies have comprehensively assessed the impact of particular manipulations on library complexity and quality, and characterized the activities and specificities of several key enzymes used in library construction. Fortunately, careful protocol design and reagent choice can substantially mitigate many of these biases, and enable reliable representation of sequences in libraries. This review aims to guide the reader through the vast expanse of literature on the subject to promote informed library generation, independent of the application.

## INTRODUCTION

Next generation sequencing technologies have undeniably changed the scientific landscape in biology. The fast-paced methodological progress driving many of the developments in the field has not only been the result of exceptional advances in sequencing chemistry, detection systems and data-processing or analysis methods (1), but also of innovations in the area of sequencing library construction. The paramount role of library construction is often underappreciated, yet it shapes both outcome and inference: the library protocol should meticulously capture the specific molecules of interest, yet minimize unwanted fragments or biases in order to ensure accurate interpretation (‘garbage in is garbage out’). Additionally, a higher quality library usually maximizes the useful sequencing read output and facilitates data processing. Indeed, in the past few years, the number of studies reporting (and in many, cases, addressing) the impact of the choice of specific enzymes, reagents, reaction conditions or overall protocols on the resulting library quality have grown exponentially, and there is renewed interest in the development of molecular biology tools designed to overcome these biases.

In addition to libraries for sequencing purposes, many proteome-wide functional assays, for instance assessing protein interactions (2,3), protein localization (4), post-transcriptional regulation (5) or drug activity (6), also rely on pooled or arrayed nucleic acid libraries as input. Fortunately, some of these libraries can now be accurately synthesized at relatively low cost, or one can rely on available collections of full-length and validated open reading frames (ORFs) on plasmids (7), short hairpin or small interfering RNA libraries (8) and guide RNA libraries for CRISPR screens (9). In several other cases, however, such as for very large libraries or libraries with custom requirements, high-quality libraries still need to be generated. Coding sequence fragment libraries are a prominent example (10–13).

Many researchers can (and do) resort to the use of commercial kits to capture the desired nucleic acid species into a workable library of molecules. While there are numerous suppliers for sequencing library construction, and the resulting libraries are often of reasonable quality for standard sequencing experiments (e.g. transcriptome sequencing), it is generally acknowledged that these conventional procedures allow little room to tailor the library toward the specific needs of the researcher, especially when the research question calls for a non-standard approach. Additionally, there is always a lag between the description of a new method and its commercialization.

The goal of this review is to provide an in-depth yet application-independent overview of current and state-of-the-art technical developments in the field, guiding the reader through the vast expanse of tools that can be used to turn a pool of nucleic acids into a library that can be sequenced or assayed using other means. We here summarized the principal insights in this fast-paced discipline, expanding on newly published studies and aspects not covered in previous reviews (14–16).

## STARTING WITH RNA

The plethora of different types of libraries all converge to dealing with either DNA or RNA (which is, eventually, almost always converted into amplifiable DNA). The starting point in RNA procedures are mostly total RNA or poly(A)^+^-RNA transcripts, but can extend to *in vitro*-transcribed (IVT) RNA, various types of non-coding RNAs, ribosome footprints, tRNAs, crosslinked RNA or modified RNA. For each of these subsets, dedicated protocols (17–23) or commercial kits exist for their purification—these are beyond the scope of this review and will not be detailed further. Nevertheless, the downstream steps for most of these molecules are generally the same.

### Ribosomal RNA depletion

Ribosomal RNA (rRNA) makes up more than 80–90% of the total RNA pool of all cells (24–26). In most applications, this large fraction is irrelevant to the question of interest. While downstream computational filtering of reads mapping to rRNA genes is always an option, these molecules take up unnecessary sequencing space, needlessly inflate screening scale when assaying libraries for expression and can reduce the overall sensitivity of the assay in question. As a consequence, rRNA depletion methods have received considerable attention, and the advantages and disadvantages of commonly used procedures are well studied.

Poly(A)-tailed RNA selection via hybridization capture using oligo(dT)-coupled beads (or variations on this theme) has been very powerful to extract protein-coding mRNA transcripts from the total RNA pool, passively depleting it from rRNA and immature or incompletely processed heterogeneous nuclear RNA (27). The most obvious downside of this method is the counterselection for all other poly(A)-negative RNAs which might potentially be of interest, many of them small non-coding RNAs transcribed by RNA polymerase III (small nucleolar RNAs (snoRNAs), several microRNAs, U6 spliceosomal RNAs, the SRP RNA component, among others) (28). The poly(A)-negative transcripts of bimorphic genes (that produce both classically poly(A)-tailed as well as non-tailed mRNAs) are also missed in this situation, which is likely the reason why their distinct roles have been overlooked for many years (29). Histone mRNAs are also known to lack a poly(A)-tail, just like the *HEG1* and *DUX* mRNAs (23), although a recent study reported the detection of 28 histone cluster genes in the poly(A)^+^ RNA fraction, arguably resulting from incorrect 3′ processing (27). Additionally, although bacteria can tag mRNAs with poly(A)-tails for the purpose of degradation (30), bacterial transcripts generally lack these tails and consequently, this strategy is not applicable in bacteria. In contrast, the 13 proteins encoded by the mitochondrial genome in eukaryotes that produce ‘prokaryote-like’ polycistronic, intron- and capless mRNAs are nevertheless also poly(A)-tailed by a mitochondrion-specific poly(A)-polymerase (27,30,31). For the purpose of rRNA depletion, poly(A)^+^ selection is effective but not complete; even after several rounds, at least 0.3% of all sequencing reads map to rRNA genes (27). Many of these rRNAs contain poly(A)-stretches in their sequence. Moreover, the enrichment for poly(A)^+^ transcripts can lead to a bias in sequence coverage through differential binding to oligo(dT), as was recently assessed by sequencing of IVT-arrayed cDNA libraries (18). Finally, for degraded RNA (especially in formalin-fixed, paraffin-embedded (FFPE) samples), poly(A)^+^ selection will only recover the 3′ portion of the transcript.

Active removal of rRNA sequences using a mixture of sequence-specific probes immobilized on beads (e.g. Ribo-Zero (Illumina) and RiboMinus (Thermo Fisher)) is a popular alternative compatible with the recovery of poly(A)-negative RNA, as it offsets many of the disadvantages of poly(A)-selection. However, remaining contaminating rRNA is also of concern, to a variable extent but generally more so than in poly(A)^+^ selection (27,32,33). Active ribodepletion using these methods can also affect sequencing coverage, especially of those genes with stretches sharing similarity with rRNA sequences (18,26). Of the most popular commercial reagents, the Ribo-Zero kit seems to be less susceptible to this coverage skewing than the RiboMinus kit, most likely because of the more stringent hybridization requirements (34). For mRNA abundance measurement in *Saccharomyces cerevisiae*, results obtained with the Ribo-Zero kit, compared to RiboMinus or poly(A)-selection, correlated the most with total RNA data (34). Enzymatic methods for active ribodepletion have also gained popularity. As such, abundant DNA sequences (like cDNAs derived from rRNAs) can be digested non-specifically using the Kamchatka crab duplex-specific nuclease (DSN) (35,36), even in a single-cell setting (37) (see below in the ‘Normalization’ section). Similarly, rRNA bound to specific DNA oligos can be digested by the heteroduplex-specific RNase H (38). Of all the common active ribodepletion methods, the RNase H method came out as overall best performer by most measures in a recent comparative study, leading to the highest rRNA depletion efficiency and the lowest coverage or GC bias, followed closely by the more expensive Ribo-Zero strategy (26). Another promising newcomer is DASH (depletion of abundant sequences by hybridization), in which ribodepletion is obtained through enzymatic digestion by recombinant Cas9 and rRNA-specific guides (39). DASH could effectively deplete mitochondrial ribosomal sequences in low-input RNA-seq libraries, reportedly outperforming several commercial RNase H-based and Ribo-Zero ribodepletion kits in performance, cost and input requirements (39).

An alternative tactic that has been used for the purpose of ribodepletion is selective random hexamer priming. By computationally subtracting rRNA-complementary hexamers from a random hexamer primer library before synthesis, the Raymond lab generated a 749 not-so-random hexamer library that could indeed selectively prime the non-rRNA transcriptome under high salt conditions (40). Leveraging the tolerance of reverse transcriptase (RT) for one or two mismatches at the priming site, the number of primers can even be reduced to below 50 while still broadly covering the transcriptome (41) and requiring only limited quantities (50 pg) of RNA with careful primer design (42). This method can also be expanded to deplete other abundant transcripts (see below in the ‘Normalization’ section) or to reduce priming artefacts (41,42). Although the selective random hexamer strategy has been used with success in RNA-seq (43), the observation that still more than 10% of reads mapped to (cytoplasmic) rRNA (40,41) makes this method much less efficient, and thus less advisable, for ribosomal depletion compared to the methods cited above.

In all, when the input RNA amount is not limiting, poly(A)^+^ selection seems on par with active ribodepletion methods like RNase H-based or DASH, and it is mostly the RNA species of interest (mRNA, non-coding RNA) that will dictate which approach is the most appropriate. However, it is important to note that none of these strategies are compatible with the minute amounts of RNA extracted from a single cell. Instead, current single-cell RNA-seq library construction methods almost exclusively rely on direct oligo(dT)-based priming (not hybridization-based physical selection) of extracted RNA to simultaneously deplete ribosomal species and prime the mRNA for reverse transcription (44–50). In one recent report, poly(A)-negative transcripts from single cells could be detected by combining oligo(dT)-priming with selective random hexamer priming and strand displacement (RamDA-Seq, Random Displacement Amplification Sequencing) (51).

### RNA fragmentation

Fragmentation is a requirement for most sequencing libraries, as uniform sizing of molecules is important for optimal performance of most ‘second-generation’ sequencing instruments. This is not only due to restrictions in read length, but also because amplification (both in solution and solid-phase) favors smaller fragments over longer ones. In addition to the observation that RNA hydrolysis is more straightforward and less prone to sequence bias than DNA fragmentation, it can mitigate some of the biases that can be introduced during the conversion to cDNA by RTs (see below). As such, RNA fragmentation reduces random priming bias during cDNA synthesis, likely by limiting secondary structure formation, and enables a more equal coverage of the 5′ and 3′ transcript ends (52).

Taking advantage of the nucleophilicity of the 2′-hydroxyl group of RNA, simple heating and addition of catalytic metal ions that act as Brønsted bases to abstract the 2′-OH proton, like Zn^2+^ or Mg^2+^, is sufficient for efficient hydrolysis (53,54). The resulting fragment ends are a mix of 5′-hydroxyl groups, 3′ phosphates, but also 2′ phosphates and 2′-,3′-cyclic phosphates (55), which can be problematic for certain downstream enzymatic steps (predominantly for RNA ligation). Consequently, such chemical fragmentation is often followed by T4 polynucleotide kinase treatment, resolving cyclic or 3′ or 2′ phosphates back to 2′ and 3′ OH groups and phosphorylating 5′ ends (56–58). Because chemical shearing is quick and efficient, and size distributions can easily be optimized by changing incubation time, it has become more widespread than mechanical methods, such as sonication, for RNA fragmentation.

Enzymatic digestion with the double-strand-specific RNase III is also an alternative, and has the advantage that it generates 5′-phosphate and 3′-hydroxyl ends more compatible with direct RNA ligation. Although the enzyme has a preference for double-stranded RNA (dsRNA), single-stranded RNA (ssRNA) can also be cleaved by modulating the salt and RNA concentration (59). However, digestion with RNase III is not completely random (60), a feature that does not really seem to affect coding region expression measurements in RNA-seq, but does substantially lead to under-representation of specific classes of non-coding RNA (61,62).

### cDNA generation

#### Reverse transcriptase

RNA requires conversion to DNA for most applications, whether it is for cloning or for sequencing. Direct sequencing of RNA has been reported (63–65) and is still an area of intense research, but is not as advanced and robust yet as the sequencing of DNA. RTs are RNA-dependent 5′→3′ DNA polymerases and can be found in all domains of life with roles in various different biological processes, although they are generally believed to have evolved from a single ancient enzyme (66). Most current commercially available RTs are derived from retroviral RTs, either from Moloney Murine Leukemia Virus (M-MuLV or MMLV), or from the Avian Myeloblastosis Virus, and show various improvements in terms of processivity, thermostability or lack of RNase H activity—factors that all affect the reliability with which RNA libraries can be converted to cDNA. Processivity issues can lead to under-representation of 5′ ends of long RNAs, such as unfragmented mRNA transcripts. Highly structured or GC-rich RNAs, such as tRNAs, are notoriously difficult to reverse transcribe, and many efforts have been directed towards increasing RT thermostability to allow for template secondary structure melting and specific primer binding at elevated temperatures (67). Modifications can also inhibit RT (68), and its RNase H activity is often undesirable as it can degrade long RNA molecules before complete cDNA synthesis has taken place, which is why several commercially available RTs have mutated RNase H domains.

**Figure 1.**
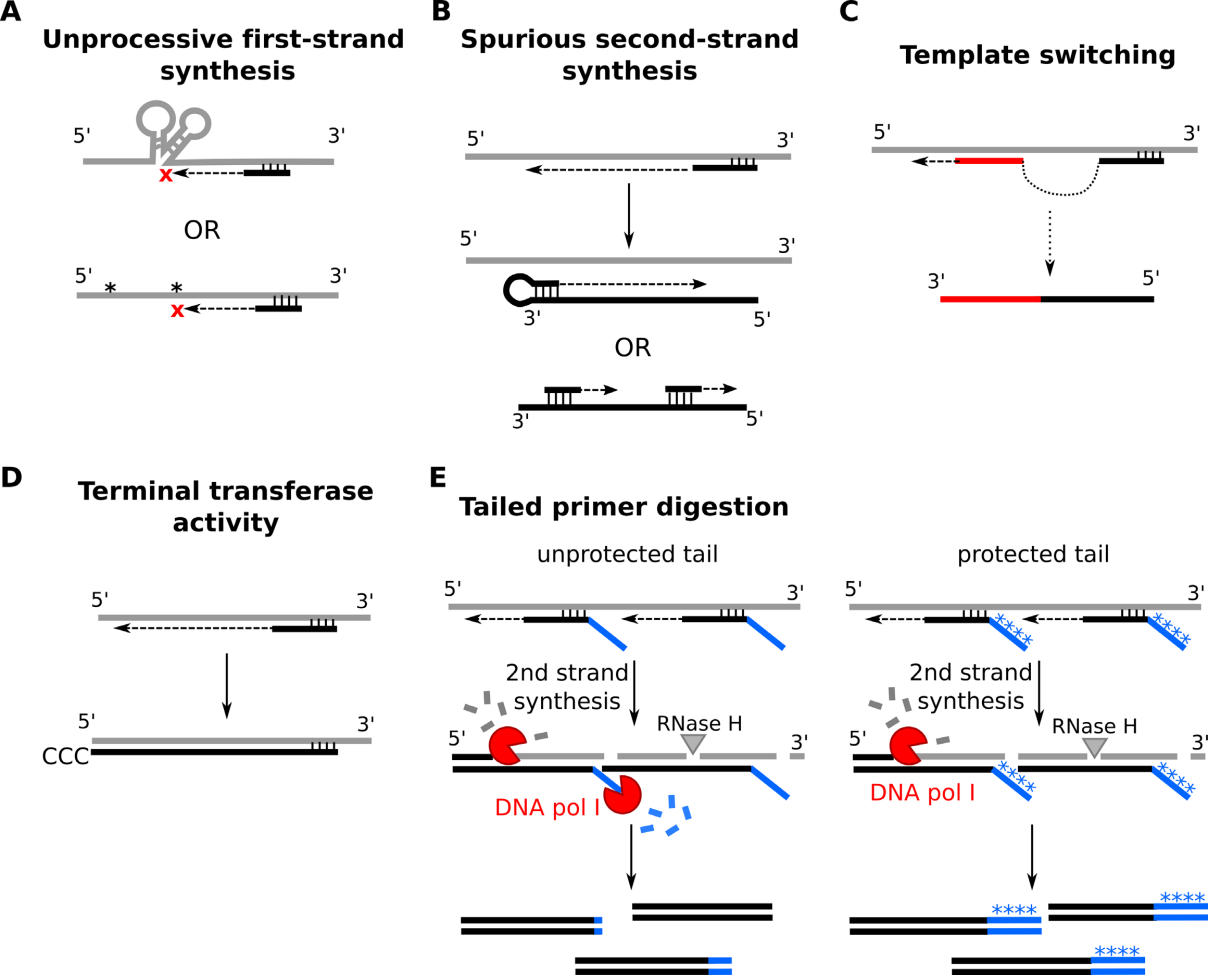
Undesired activities during cDNA synthesis. (**A**) The processivity of retroviral RTs is generally limited, which is problematic for complete reverse transcription of long RNAs. Secondary structures (gray) or modifications like 2′-O-methylation (indicated by *) in the RNA template can further impede full retrotranscription. Black = cDNA strand with annealing primer (random, oligo(dT) or specific). (**B**) Artefactual antisense products can be formed due to DNA-dependent DNA polymerase activity of RT during first-strand synthesis. This can occur through looping or repriming of the first cDNA strand. (**C**) During template switching, the RT repositions itself (and the synthesized first cDNA strand) further downstream of the same template, or a new one, during synthesis, leading to gapped synthesis of cDNA of intra-molecular fusions. (**D**) MMLV RTs have terminal transferase activity with a preference for template-independent cytosine addition. (**E**) cDNA synthesis with tailed primers. If the tail (blue) is unprotected, the Y-bifurcation formed is susceptible to the nuclease activity of DNA polymerase I during second strand synthesis, leading to incomplete incorporation in the final product. This can be mitigated by including phosphorothioate bonds or buffering bases (*) in the primer tail.

Despite these efforts, however, reverse transcription remains a significant source of bias during library generation. A principal aspect of all RTs is the intrinsic lack of 3′→5′ exonuclease or ‘proofreading’ activity. Error rates are high compared to DNA polymerases, and vary between 1/9000 and 1/30000 depending on the assay and enzyme, compared to 10^−6^–10^−8^ for DNA polymerases (69–71). While this is less of an issue for small RNA library construction, and can be mitigated in sequencing library construction by including more technical replicates, it remains difficult to analyze RNA sequence polymorphisms (72,73) and can be problematic in assays that rely on expression of the molecule. In addition to the RT’s low processivity (Figure 1A) and relatively high error rate, several artefactual activities have been reported as well. As such, intrinsic DNA-dependent DNA polymerase activity can lead to spurious second-strand DNA during first-strand synthesis, leading to artificial antisense sequences (64,74–76) (Figure 1B). Reportedly, the addition of actinomycin D, which binds deoxyguanosines, can suppress this activity (77,78). Template switching, in which the RT and cDNA dissociate from the RNA template and reanneal to a different stretch, creates chimeric sequences, false deletions and inexistent splice variants (79,80) (Figure 1C); 1–7% of all reads show evidence for this phenomenon (64). MMLV RTs are known to add additional bases at the 3′ end of the newly synthesized cDNA strand (81) (Figure 1D). The latter feature has been turned into an asset in some cDNA synthesis protocols, such as the SMART (switching mechanism at the 5′ end of the RNA template) method, in which the dC tail preferentially appended by RT is used for hybridization with an oligo(G)-containing primer for second strand synthesis (82). However, this terminal transferase activity of RTs is undesired in expression libraries as the extra bases could interfere with the reading frame and could result in proteins with extra amino acids. Finally, MMLV-derived RTs can be sensitive to 2′-O-methyl modifications in RNA (83) (Figure 1A), which can be an issue for mammalian piwiRNA or plant microRNA reverse transcription (84).

**Figure 2.**
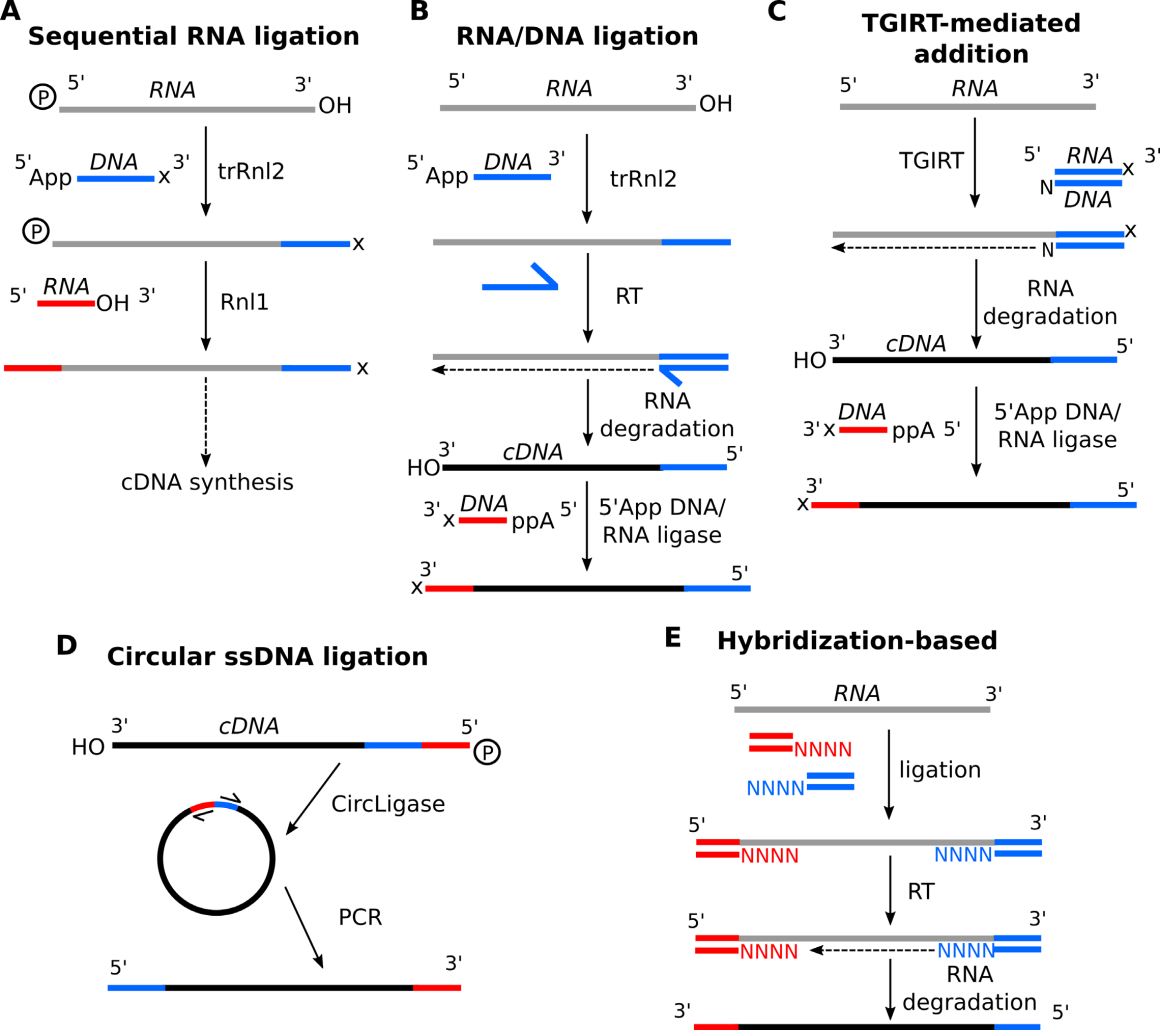
Common strategies for RNA adaptor ligation. (**A**) RNA substrates with 5′ phosphates and 3′-OH can be sequentially ligated with a 5′ pre-adenylated (App), 3′ blocked (x) DNA adaptor using truncated Rnl2 (ideally the K227Q R55K mutant), and a 5′ unphosphorylated, 3′ hydroxylated RNA adaptor with Rnl1. Sometimes the primer for reverse transcription is added before 5′ adaptor ligation. (**B**) In RNA/DNA ligation, RNA substrates with 3′-OH are ligated to a 3′ adaptor as in A, but no blocking is required. After reverse transcription by RT and degradation of the RNA strand, the 3′-OH of the resulting cDNA strand is ligated to a 5′ preadenylated, 3′ blocked DNA adaptor using the 5′ App DNA/RNA ligase (Mth K97A). (**C**) In TGIRT-mediated addition, RNA templates are immediately reverse transcribed and adaptor ligated via TGIRT and a double-stranded, single random overhang adaptor. Ligation of the other adaptor can be done as in B. (**D**) CircLigase can be used to circularize single-stranded cDNA molecules that were ligated to a bifunctional adaptor on one side using either RNA ligation or TGIRT-type methods, followed by reverse transcription. After circularization, the adaptor can serve as starting point for PCR to regenerate linear molecules with a different adaptor on both sides. (**E**) In hybridization-based RNA ligation, RNA templates are ligated to adaptors with randomized single-stranded overhangs, and then reverse transcribed.

Two recent promising developments deal with several of these issues at once. The first has come forth from the study of maturase RTs, an alternative class of RTs found in non-long-terminal-repeat retrotransposons (66) and in intron-encoded proteins of group II introns (85). The Lambowitz group focused on bacterial mobile group II intron RTs, which have evolved to reverse transcribe very structured group II intron RNAs (86). Known as TGIRTs, or Thermostable Group II Intron RTs, these RTs have higher thermostability, higher processivity and about 2-fold higher fidelity than the commercial golden standard retroviral RTs (SuperScript III) (86). They can also read through modified bases, and while the template switching frequency remains the same (about 0.14% of reads), the resulting deletions are only rarely internal (87). The authors also discovered that RNA–DNA duplexes with single 3′ N-overhangs can be used to directly couple the cDNA strand to an adaptor sequence (86,87) (see also Figure 2C). The method has been broadly adopted, also for the sequencing of highly structured tRNAs (21,88–90). Another exceptionally processive and highly soluble maturase RT was recently discovered in *Eubacterium rectale* (91). While this ‘MarathonRT’ remains to be validated in a next-generation sequencing context, the observation that it can reverse translate a 5 kb transcript with less background than TGIRT make it especially promising for long-read sequencing technologies such as PacBio (92).

A second advancement, reported by the Ellington group, is the modified direction evolution of a high-fidelity thermostable DNA polymerase to enable reverse transcription with proofreading (71). The final reverse transcription xenopolymerase (RTX) has a 3- to 10-fold lower error rate than MMLV RT (3.7*10^−5^ versus 1.1*10^−4^), remains thermostable and processive, and was shown to be completely compatible with RNA-seq, leading to nearly identical coverage and expression profiles as an established RT (71).

#### Priming

RTs require a primer for first strand cDNA synthesis. Unless a sequence-specific primer can be used (e.g. in the case of TGIRTs or after RNA ligation, see below), the standard approach relies on either oligo(dT) or random primers. Homopolymer stretches, mostly poly(A), can be added to substrates without poly(A)-tail to enable oligo(dT) priming (93). The *Escherichia coli* poly(A) polymerase, the most often used tailing enzyme in these approaches, is however significantly affected by terminal stemloop structures (94,95) and to a lesser extent by 3′ nt identity (84) of the substrate, although both features can be minimized by adapting reaction conditions (increased temperature and reaction times). Nevertheless, the addition of bases can be problematic if the products are to be cloned for expression downstream in the procedure, as it may disrupt the frame or add unwanted codons. Poly(A)-tailing can also obscure the identity of the 3′ base of each template fragment, as an original 3′ adenosine may be mistaken for the synthetic poly(A)-tail. Moreover, as most vertebrate piRNAs and plant miRNAs carry 2′-O-methyl groups at their 3′ ends instead of 2′-OH (96,97), and these ends are poor substrates for poly(A) polymerases (84), the method is not suited to capture these types of RNAs.

A frequently used alternative is random priming. Primers as short as 6 bp are capable of sequence-specific RNA binding (98). Consequently, for random priming, random hexamers or heptamers are most commonly employed. In comparison with oligo(dT) priming, the random approach was shown to enable more equal sequence coverage across mRNA transcripts in early RNA-seq studies, especially after RNA fragmentation attenuate structure formation (52). Nevertheless, random primer annealing is prone to skewing; one meta-analysis of several RNA-seq experiments revealed that nucleotide frequencies of the 13 first nucleotides of each read were clearly diverging from the expected 1:1:1:1 A:C:G:T ratio in a manner that correlated with the type of primer used (random or not) (99). While there is a role for thermodynamic preferences toward GC-rich sequences, the actual skew depends on the composition of the transcriptome and also on motif preferences of the exact RT and polymerase used during cDNA synthesis (99,100). This positional bias can be corrected for *in silico* (99).

Simple random priming does not retain strand information, however. To do so, it is possible to tag random primers (or oligo(dT) primers after fragmentation) with specific sequences (and for instance, add a restriction site or barcode). These tails reportedly only modestly influence priming (40,100,101), although a rigorous systematic assessment is lacking. It is important to note that these non-hybridizing tags of random primers are sensitive to nucleolytic degradation, which can lead to inactivation of incorporated restriction sites and loss of directionality (100–102) (Figure 1E). This phenomenon has been attributed to the 5′→3′ exo- and endonuclease activity of DNA polymerase I during second strand synthesis, which has a particular preference for single-stranded DNA (ssDNA) in bifurcated duplex structures (103,104). The incorporation of nuclease-resistant phosphorothioate bonds (100) or additional bases that buffer the tag sequence (101) can counter this effect. Alternatively, the DNA polymerase I can be replaced by the 5′→3′ exo- Klenow fragment, a proteolytic product of the *E. coli* DNA polymerase I which only retains polymerase and 3′→5′ exonuclease activity, but this requires the availability of a second primer binding site for second strand synthesis and full degradation of the RNA template (40).

How sensitive are these methods for the generation of single-cell libraries? As alluded to above, the greatest strength of oligo(dT)-based priming is its ability to combine ribodepletion and priming of mRNA for reverse transcription in a single step, which is why this strategy has become by far the most widespread starting point for single-cell transcriptome library synthesis (44–50). The Huang lab has however shown that tagged random priming can also be accommodated to minute input amounts without massively amplifying rRNA; the authors speculate that the mild lysis conditions and specific reverse transcription procedure likely contribute to this effect (105).

#### RNA ligation

A popular alternative to oligo(dT) or (tagged) random primers is the ligation of adaptors at the RNA level prior to cDNA synthesis. Crucially, this method preserves the directionality of RNA molecules and is thus a stranded approach, provided that the necessary end groups are protected. Combined with an rRNA-masking oligo, RNA ligation can also be used in a single-cell setup (106).

In general, single-stranded adaptors are sequentially ligated, first to the 3′ end of the RNA molecule, and before or after cDNA synthesis, to the other end (107) (Figure 2A and B). In order to avoid domination of circular or concatamerized products, without having to resort to extensive dephosphorylation/rephosphorylation reactions, most protocols rely on a C-terminally truncated form of T4 RNA ligase 2, trRnl2, which has lost the ability to use free adenosine triphosphate (ATP) to catalyze ligation reactions (108). Using pre-adenylated DNA adaptors (App-adaptor) (Figure 2A and B), free 3′-OH RNA ends can be adaptor ligated, effectively avoiding circularization (109). Adaptor–adaptor concatamers are avoided as the enzyme requires 3′ RNA, not DNA, ends, although in practice, 3′ adaptor ends are nevertheless often blocked (e.g. –NH_2_, three-carbon or six-carbon spacers) for the 5′ ligation reaction. The trRnl2 does tend to deadenylate the App-adaptors and to subsequently adenylate the substrate RNA molecule, leading to substrate concatamers and circles; the K227Q point mutant lacks this activity, leading to less side products (110). The mutation does slightly affect ligation efficiency, but this has been mitigated using a compensatory R55K mutation (leading to ‘trRnl2 K227Q R55K’). A related pre-adenylation dependent enzyme, Mth K97A, derived from the *Methanobacterium thermoautotrophicum* RNA ligase, has the added advantage of thermostability, facilitating the melting out of potentially inhibitory RNA structures in the template (111). The enzyme does show a preference for A and C at the third nucleotide from the ligation site (112).

After 3′ adaptor ligation, the 5′ adaptor can either be ligated to the 5′ end of the RNA before first strand synthesis, or to the 3′ end of the resulting cDNA strand after first strand synthesis. In the former scenario, the RNA substrate 5′ phosphate is linked to the 5′ RNA adaptor's 3′ hydroxyl by the ss T4 RNA ligase 1 (Rnl1) (113) (Figure 2A). To avoid side products, the substrate’s 3′ end should be blocked, and the adaptor should not be phosphorylated at the 5′ end. As the Rnl1 is much more a single-strand specific ligase than Rnl2, often the DNA primer for reverse transcription, which anneals to the 3′ adaptor, is added even before the 5′ adaptor ligation step. This also reduces undesired products caused by excess unligated 3′ adaptor.

Alternatively, the 5′ adaptor can be ligated to the first strand cDNA after degradation of the RNA strand, for instance through alkaline treatment (69) or RNase H digestion (Figure 2B). Provided that the 5′ adaptor (DNA) is 5′ adenylated and 3′ blocked, the ATP-independent thermostable Mth K97A (sometimes referred to as the 5′ App DNA/RNA Ligase) is used for this, as it has better ssDNA ligation activity than (tr)Rnl2 (111).

Both 3′ and 5′ end RNA (or ssDNA) ligation biases are significant and have been extensively documented, mostly in the context of small RNA sequencing (72,73,95,112,114–117). Using synthetic equimolar pools of more than 900 different miRNAs, the Brett Robb lab measured that differences in ligation efficiencies between single molecules can introduce up to 10 000-fold abundance variation, independent of polymerase chain reaction (PCR) biases (118). Although initially, this bias was often attributed to primary sequence preferences, it has become clear that the structural properties of the RNA substrate, the adaptor and the propensity of substrate and adaptor to form stimulating or inhibitory ‘cofold’ structures, control the efficiency of ligation at both sides, although the role of different structure classes differ for 3′ end and 5′ end (72,73,118). An exhaustive investigation has further revealed that careful adaptor design can substantially suppress these issues (118). As such, ideal 5′ and 3′ adaptors contain a degenerate, randomized middle sequence portion (6 nt), which does not have to be adjacent to the ligation site, to ensure flexibility in generating favorable ligation structures. Additional bias reduction can be obtained by including short (7 nt) complementary stretches between the 3′ and 5′ adaptor, as these hybridized adaptor structures stimulate ligation (118).

Alternatively, to avoid the biases associated with 5′ end ligation by Rnl1, 3′ adaptor-ligated products (with 5′ phosphates and 3′ OH, no 3′ blocking) can be reverse transcribed as per usual, but then circularized by a pre-adenylated ssDNA ligase (‘CircLigase’) and PCR amplified (Figure 2D). This CircLigase strategy has been used successfully for ribosome footprint capture and the sequencing of DMS-treated RNA for structure probing (119,120), and can indeed reduce, though not completely abolish, the over-representation of particular sequences (112). A comparison of several RNA-seq library prep methods indicated CircLigase as the method that resulted in the most uniform coverage (121). The circularization efficiency, however, reportedly decreases for longer cDNAs (87), and is less suited for pools of molecules with a broader size range. Another option is to ligate with splinted adaptors—double-stranded adaptors containing single-stranded degenerate overhangs to the RNA molecule (122) (Figure 2E). Note that since splinted adaptors contain a random portion for hybridization, a GC-bias is expected and imperfect annealing will inhibit ligation (123).

RNA ligation can be a challenge when substrate RNA molecules are modified at their 5′ or 3′ ends. Under the right conditions, 2′-O-methyl groups are not an issue for trRnl2 (84). In contrast, 3′ end 2′, 3′-cyclic phosphates are not ligatable. For resolution of unwanted 2′, 3′-cyclic phosphates, as arises after divalent cation or ribozyme, RNase A, RNase T1 or RNase 1 activity, treatment with wild-type T4 polynucleotide kinase in acidic conditions is sufficient, as mentioned before. For 5′ end ligation of RNA molecules that lack a regular 5′ phosphate, enzymatic treatment with tobacco acid pyrophosphatase to remove cap structures or with T4 PNK to phosphorylate 5′-OH ends, can be necessary (123,124).

#### Second strand synthesis

Second strand synthesis is generally performed using the very efficient and versatile classical Gubler and Hoffman method (125), or one-tube versions that are offered commercially. Principally, the method combines *E. coli* RNase H digestion, which creates nicks in the RNA strand of the RNA–DNA duplex after first-strand synthesis, *E. coli* DNA pol I, which can use these nicked sites as primer for 5′→3′ DNA synthesis while displacing and degrading the RNA in the same direction through its 5′→3′ activity, and *E. coli* DNA ligase, which ligates the nicks. Overhangs are degraded through the 5′→3′ and 3′→5′ nuclease activities of the DNA pol I, leaving blunt ended DNA.

Although this classical Gubler and Hoffman second strand synthesis method is not intrinsically strand specific, the polarity of transcripts can be retained by replacing dTTP with dUTP in the second strand synthesis reaction. The introduction of uracil blocks high-fidelity amplification of the second strand in the PCR step (126), and combined with the appropriate adaptors (see below), all amplified molecules will consequently have the same orientation. Alternatively, the uracil-containing strand can be degraded using a mixture of uracil–DNA glycosylase and DNA glycosylase-lyase endonuclease VIII (NEB’s USER) before PCR. The method is popular and efficient, and it performed best among several other strand-specific methods for RNA-seq with regard to a variety of criteria, including evenness of coverage and strand specificity (127).

If specific sequences are incorporated prior to second strand synthesis, for instance through RNA ligation or SMART-type template switching, double-stranded DNA (dsDNA) can be generated from the single-stranded cDNA through PCR amplification. This approach is sensitive and suitable for second strand generation in single-cell setups (47,48,106).

## STARTING WITH DNA

In many applications, DNA is the starting point of the library synthesis. This can be genomic DNA, immunoprecipitated DNA such as in ChIP (chromatin immunoprecipitation) or MeDIP (methylated DNA immunoprecipitation), targeted sequence captured DNA or any other method where a specific subset of sequences requires library synthesis. Alternatively, existing DNA collections such as the human ORFeome (7) can also be used as source material. Several fragment libraries for yeast-two-hybrid screening have been constructed from such collections, by PCR amplification of ORFs and titrated exonuclease digestion for progressive removal of vector end sequences (13,128).

### DNA fragmentation

DNA fragmentation is required for short-read sequencing library construction when starting from molecules longer the required platform range. Additionally, fragmentation is also an intrinsic part of fragment library generation for expression or protein–protein interaction screening. Compared to RNA, the double-stranded configuration and lower reactivity of the deoxyribose in DNA makes it more difficult to hydrolyze. Hence, one generally resorts to physical shearing methods using sonication, nebulization or acoustic shearing; or to enzymatic methods.

With sonication or nebulization, the size range tends to be wide and difficult to adapt, resulting in low yields; sample heating in the process may additionally lead to DNA damage and strand dissociation (129–131). The Covaris method of focused acoustics is considered best-in-class, with low sample loss, tunable DNA size ranges and high reproducibility (130). Fragmentation using either of these three methods nevertheless results in the preferential cleavage at CG dinucleotides (132), suggesting this is perhaps a typical attribute of physical shearing of DNA. Whatever the origin, this preference thus introduces a form of bias at an early step in the procedure.

Early reports (from 2006) employing DNase I digestion to randomly fragment DNA described the method as essentially bias-free (133,134). The DNase I endonuclease is often used in DNase hypersensitivity assays for chromatin analysis, and in transcription-factor footprinting methods. However, closer inspection of several hypersensitivity sequencing datasets revealed a clear preference for sites with cytosines at the −2 position of the cut site (135). The latest generation of fragmenting enzymes or enzyme blends (such as the NEB Fragmentase, with a nicking enzyme and an endonuclease cleaving the opposite strand) perform well in comparison, being less susceptible to sequence bias (136) and giving more consistent results than sonication or nebulization (137). Size range can easily be customized by modifying the DNA-to-enzyme ratio and digestion time, and as the resulting products are blunt ended, no end repair step is needed downstream.

Random priming of DNA material has been done as well (138). While short random hexamers and heptamers give satisfactory results for RNA, longer primers are required to offset competition for annealing with the opposite strand when working with dsDNA (139). The incorporation of a hairpin structure in the 5′ portion of the random primer has been reported to substantially reduce the number of byproducts due to random primer self-annealing in ChIP-seq libraries (140). Nevertheless, the strategy is far from ideal for the generation of random fragments, as it tends to be less efficient and more sequence-biased than other methods.

Methods in which uracil is doped into the DNA to enable fragmentation have been popular for protein fragment expression screening (141). Amplicon libraries can be amplified in a PCR with the regular four dNTPs and low amounts of dUTP. Fragmentation can then be induced at the doped sites, by uracil–DNA glycosylase digest for abasic site generation, nicking at these sites by the apurinic/apyrimidinic endonuclease IV and the generation of a double-strand break by the cleavage of the strand opposite the nick by S1 nuclease (10,142). Others have used a combination of endonuclease V and Mn^2+^ to induce double-strand breaks after uracil doping (143,144). The size distribution of the fragments can be manipulated by modulating the dUTP/dTTP ratio (10). Note that using this strategy, AT-rich regions will be more prone to cleavage compared to GC-rich regions, as more break-inducing dUTPs are incorporated (144).

### Adaptor ligation to DNA

**Figure 3.**
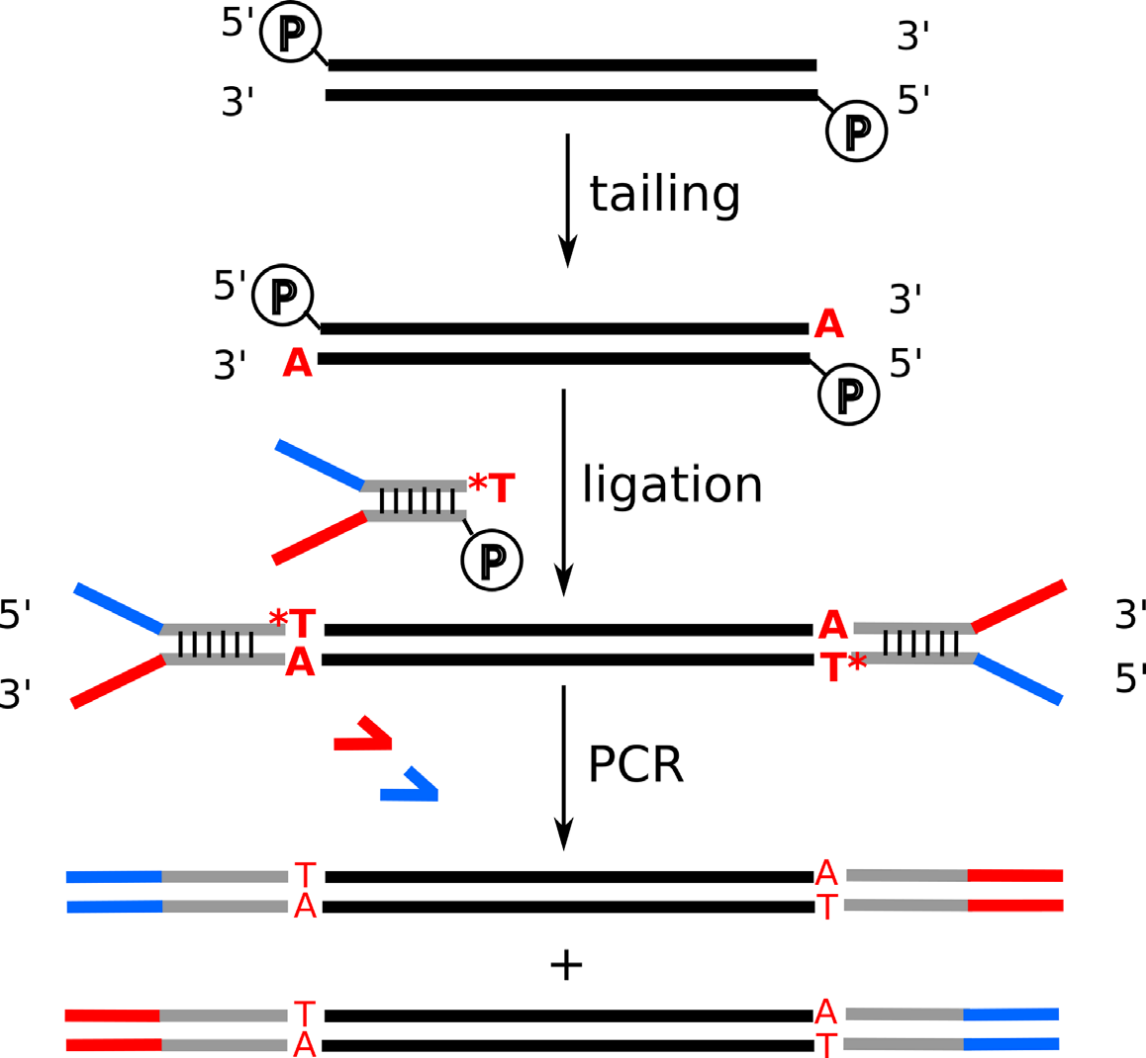
DNA template ligation with Y-shaped adaptors. Blunt-ended dsDNA templates (5′ phosphorylated and 3′-OH) are tailed at the 3′ of each strand, typically with single adenosines using Klenow fragment. Semi-single-stranded, Y-shaped adaptors with single 3′ T overhang and 5′ phosphorylation at the duplex can then efficiently be ligated. A PCR step enables the generation of molecules with different adaptors on both sides, although strand information is not intrinsically kept using this procedure. * = phosphorothioate bond.

Depending on the fragmentation method, in most cases, ends of dsDNA need to be repaired or ‘polished’ to blunt ends before downstream processing. Polishing involves digestion with enzymes that fill in 5′ overhangs and remove 3′ overhangs; T4 DNA polymerase (sometimes combined with Klenow fragment) is mostly used for this purpose (145). Generally, this is combined with T4 polynucleotide kinase to phosphorylate 5′ ends that lack phosphates. To ligate the adaptors, ultrapure T4 DNA ligase preparations can also boost ligation efficiencies (130). The most popular adaptor design combines template phosphorylation and 3′ tailing with a single nucleotide (usually A, although G-tailing is efficient as well), followed by ligation with a single T (or C) -tailed, Y-shaped adaptor (146) (Figure 3). This combination maximizes the ligation efficiency by avoiding blunt-end ligation, while effectively sidestepping template concatamerization and adaptor dimer formation. Indeed, the number of artefactual products produced through blunt-end ligation of adaptors in the original protocols for PacBio sequencing library preparation can be substantially reduced by simply switching to A/T ligation (BioRXiv: https://doi.org/10.1101/245241). Y-shaped adaptors have the added advantage that molecules in the library are tagged with a different adaptor sequence on the 5′ and 3′ end (Figure 3). For extra nuclease protection, phosphorothioate bonds are often added at the single-stranded adaptor ends (146). For sequencing on Oxford Nanopore platforms, one strand of the Y-shaped adaptor, with the so-called leader sequence, is functionalized with a motor protein to pull the DNA through the pore, and the other is hybridized to a tether to concentrate the molecule on the membrane surface (147). A variation on the Y-shaped theme is the hairpin or stem–loop adaptor, which is used in several commercial kits for next-generation sequencing library preparation (e.g. NEBNext Illumina adaptor, PacBio hairpin adaptors and Oxford Nanopore hairpin adaptors). Primer binding for amplification or sequencing is possible when the loop is large and unstructured enough (as in the PacBio adaptor), or by introducing a single uracil in the hairpin loop (as in the NEBNext Illumina adaptor), such that the loop can be cleaved using a mix of uracil–DNA glycosylase and DNA glycosylase-lyase endonuclease VIII (also referred to as ‘USER’).

Uracil-containing adaptors have been useful in various other alternative approaches for DNA adaptor ligation. The DLAF (directly ligate adaptors to first-strand cDNA) method for ligation of adaptors to ssDNA (e.g. first-strand cDNA) uses double-stranded ‘splint’ adaptors containing single-stranded overhangs of five to six random nucleotides for hybridization-based ligation with T4 DNA ligase (148). As the strand with the overhang is doped with deoxyuridines, USER treatment can degrade that strand after ligation and the resulting single-stranded adaptor-ligated DNA can be amplified (148). In another example, commercialized by Swift Biosciences, dsDNA is ligated to the individual strands of the Y-shaped adaptor in a sequential reaction (149). In the first ligation, a semi-single stranded 3′ blocked adaptor is ligated to one strand only of the dsDNA molecule. USER treatment can then degrade the non-ligated strand due to the presence of deoxyuridines, consequently allowing the next adaptor strand to anneal and ligate (149). In a third example, a combination of dUTP-doped forward and regular reverse primers can be used to amplify DNA, and USER treatment asymmetrically releases one strand of one of the adaptors on the molecule, which is then ligated to a 5′ blocked single-stranded oligo (150). This ‘reshaping’ of adaptors on DNA has been used to resolve problematic instances of intramolecular hairpin formation due to adaptor complementarity, which precludes Ion Torrent sequencing (150).

The ligation-based schemes with the Y-shaped or hairpin adaptors mentioned above are efficient, and the formation of side products is strongly reduced. Nevertheless, the procedure requires much sample-handling and is incompatible with very limited inputs (e.g. DNA from single cells). In contrast, the clever ‘tagmentation’ approach, which uses an engineered hyperactive Tn5 transposase for simultaneous DNA fragmentation and tag (or adaptor) insertion, is fast and suited for low input amounts (151). A general point of concern for tagmentation, however, is insertion bias. Although negligible for DNA sequencing of human genomes, the skews are significant in GC-rich, small genomes or when using PCR products as a starting material (151,152).

More difficult input sample types require adapted protocols. Highly degraded DNA, especially from ancient or FFPE samples, has a higher proportion of ssDNA and the input material is often only available in trace amounts. Single-strand compatible methods include the Swift Biosciences approach of sequential ligation as outlined above (149), but tailing of the ssDNA to enable priming and dsDNA generation has also been used (153,154). The Meyer lab has developed a method based on ssDNA ligation of single-stranded biotinylated adaptors using CircLigase, which avoids loss of material during purification as the sample is bound to streptavidin-coated beads (155). A recently improved version of this approach, ‘ssDNA2.0’, replaces the adaptors with splinted adaptors and the ligase with T4 DNA Ligase, and was shown to be superior for ancient DNA sequencing library preparation (156).

### Capturing methylation

Analyzing the methylation status of the genome requires the construction of libraries of methylated DNA. The golden standard for genome-wide profiling of 5′-methylcytosines (5mC), the most established DNA methylation mark, relies on chemical treatment of (generally fragmented) DNA with bisulfite (157). Bisulfite deaminates unmethylated cytosines (C) to uracils (U) while leaving 5′-methylcytosines intact (158). As such, comparing bisulfite-treated and untreated samples reveal loci with unconverted, and hence methylated, cytosines. While powerful, the use of bisulfite has several important repercussions. First, efficient amplification of bisulfite-treated DNA requires a polymerase that can tolerate the presence of unnatural deoxyuridines, and cope well with the now more abundant AT-rich regions (see section ‘Amplification’). The current best performer in that regard is considered to be the KAPA HiFi Uracil+ DNA polymerase (BioRxiv: http://dx.doi.org/10.1101/165449), which has a mutated uracil-binding pocket to avoid stalling at uracils. Second, bisulfite treatment can also result in the loss of cytosine bases and subsequent DNA breakage at the resulting abasic sites, consequently inducing DNA fragmentation (159). As this especially affects regions of unmethylated C-rich sequences, this can significantly skew sequence representation and estimation of methylation levels, although a reduction of denaturation temperatures and bisulfite concentration can limit these effects (BioRxiv: http://dx.doi.org/10.1101/165449).

The ligation of adaptors is therefore also not arbitrary in bisulfite protocols. Because of the aforementioned degradation issue with bisulfite, pre-bisulfite ligation (160,161) leads to sequence bias (BioRxiv: http://dx.doi.org/10.1101/165449) and requires relatively high input amounts. In addition, it necessitates adaptor synthesis with full cytosine-to-5′-methylcytosine replacement in order to avoid uracil conversion of the adaptor (160,161). The more recent post-bisulfite ligation strategies exploit bisulfite-induced degradation for fragmentation and only attach adaptor sequences after bisulfite treatment, for example using random primer extension (post-bisulfite adaptor tagging or PBAT) (162–164) or hexamer-guided partially single-stranded adaptors (SPlinted Ligation Adaptor Tagging—SPLAT) (165). These methods are substantially less bias-inducing compared to pre-bisulfite ligation (BioRxiv: http://dx.doi.org/10.1101/165449) and have pushed the starting material limit down to the nanogram and even single-cell (163,164) range.

Although the above whole-genome bisulfite sequencing methods allow for full genome-scanning of methylation status, only a fraction of the genome is generally (differentially) methylated, and it can be more efficient and cost-effective to focus on methylome-relevant regions instead of whole genomes. One strategy involves the digestion of genomic DNA with methylation-insensitive restriction enzymes that recognize CG-rich sites, such as CCGG in the case of MspI, thereby enabling enrichment of regions with high CpG content. Combined with bisulfite treatment of digested and size-selected fragments, such reduced bisulfite representation sequencing (RRBS) allows the monitoring of a reproducible subset of CpG islands in genomes (166,167). Enrichment for certain sites can be modulated through careful selection of the restriction enzyme (168). Although powerful and amenable to single-cell studies (169), all RRBS methods are currently critically depend on some form of size selection to maximize their enrichment factor, and thus are incompatible with highly fragmented circulating cell-free DNA (170,171). Further innovations in RRBS protocols will address these limitations (De Koker *et al.*, in preparation).

Alternatives to bisulfite-based strategies focus on pulldown of methylome-relevant regions using methyl-binding domains (172,173) or 5mC-binding antibodies (174). These methods, however, require more input DNA than PBAT, SPLAT and RRBS, and do not have single-basepair resolution of methylation status.

## AMPLIFICATION

Although PCR is an extremely powerful technique, it is well known that the amplification of pools of molecules with different sequences and lengths, as occurs in libraries, can result in serious distortion of relative abundances, with under-representation or over-representation of particular sequences. Extremely GC-rich or GC-poor templates are generally difficult to amplify, while short sequences are preferentially amplified. Stochastic effects account for part of the bias as well (175). Additionally, errors can accumulate in templates, often at low-complexity regions, and side products resulting from overamplification, such as concatamers or self-primed chimeric sequences (176), are common. However, the extent of these issues can be attenuated by careful optimization of PCR conditions and polymerase choice. For instance, the monitoring of PCR cycle number to remain in the exponential phase was shown to substantially reduce the number of overamplification products (177–179) and to reduce effects of bias toward shorter sequences (180). Carrying out the reaction on beads in emulsion (emulsion PCR) also reduces the number of chimeras, as single molecules are amplified in individual compartments, which reduces cross-priming (181). The addition of compounds such as betaine can largely prevent the under-representation of GC-rich templates, but it does not improve bias against AT-rich sequences (182). The opposite is true for TMAC (tetramethyl ammonium chloride) (183). Aside from PCR cycle number, the biggest impact comes from the polymerase used. Quail *et al.* systematically compared polymerase performance for sequencing library amplification over a range of different contexts, revealing considerable differences in fidelity, yield, sequence-sensitivity and processivity between the 23 polymerases tested (184). The KAPA HiFi enzyme, engineered for increased affinity towards DNA via directed evolution, came out as best performer, as it has the unique ability to amplify the most difficult (AT- or GC-rich) templates. The sequencing results of pools amplified by KAPA HiFi closely matched those of PCR-free libraries (184). The KAPA polymerase also surpassed the acclaimed Q5 high-fidelity polymerase (NEB), whose processivity has been enhanced through fusion with an additional DNA binding domain, in terms of accuracy and proportion of chimeric molecules (185). However, this high fidelity may come at a cost: the authors of the latter study also observed the surprising ability of both KAPA and Q5 enzymes to edit primer sequences (4% of primed molecules), leading to the unwanted amplification of sequences with small primer mismatches.

It is possible to generate libraries without the need for amplification, although the high sample input amounts (up to 5 μg) limit the breadth of applications of such amplification-free methods. The Turner lab demonstrated the superiority of PCR-free sequencing library construction using simple ligation of Y-shaped adaptors that contain all the necessary sequences required for Illumina sequencing, in the sequencing of extremely AT- or GC-rich bacterial genomes (186). Similarly, adaptor-ligated RNA libraries do not have to be amplified for RNA-seq using the FRT-seq method (Flowcell Reverse Transcription Sequencing), in which reverse transcription is performed on the Illumina flow cell prior to bridge amplification and sequencing (187).

**Figure 4.**
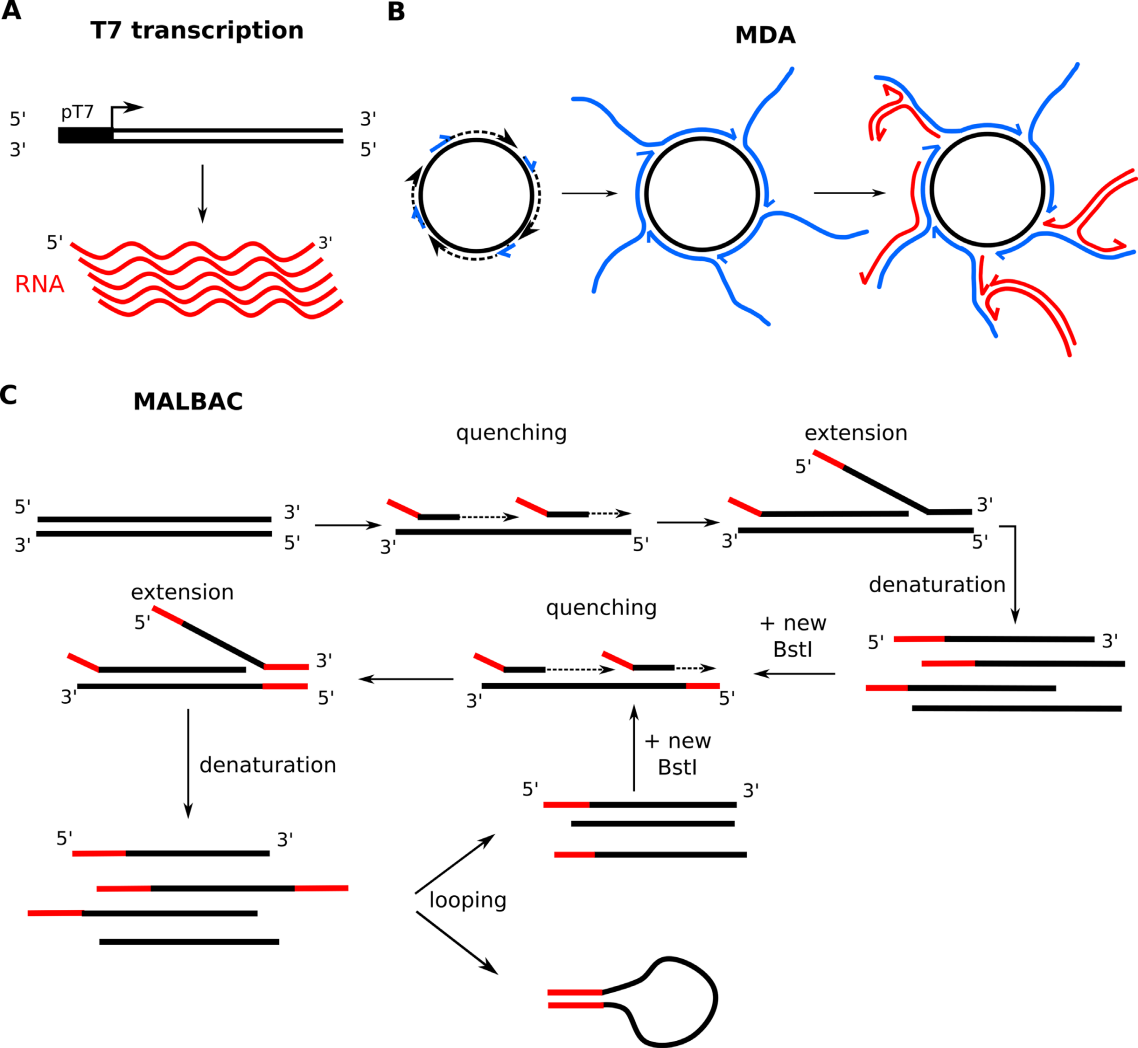
Linear and semi-linear methods for amplification. (**A**) DNA molecules tagged with a T7 promoter sequence (e.g. in the adaptor), T7 RNA polymerase-based transcription can be used for amplification. (**B**) MDA involves (random) priming of linear or circular molecules and isothermal amplification with a strand-displacing enzyme such as the ϕ29 polymerase. The displaced strands can be used for multiple new rounds of priming and displacement (red). (**C**) MALBAC amplification involves priming of molecules with tagged random primers at low temperature (quenching), strand displacement amplification with BstI (extension) at 65°C, and denaturation. The cycle is repeated with fresh enzyme. Molecules with two tail sequences, which is the desired end product, accumulate during each cycle, but are not further amplified as their tails associate. After several cycles, the sample is enriched in molecules with tags on both sides, and can be amplified further via PCR.

However, when the input material is limited, such as in the extreme case of single-cell sequencing, many researchers resort to (semi-)linear amplification methods to amplify the material while minimizing artifacts. Because of the exponential aspect of PCR, errors quickly propagate and biases are exacerbated; this cumulative effect is less extreme for linear methods relying on the T7 RNA polymerase or strand-displacing enzymes such as the BstI or ϕ29 polymerases. The bacteriophage T7 RNA polymerase methods rely on *in vitro* transcription of DNA molecules encoding a T7 promotor, a system routinely used for microarray sample preparation (188,189) (Figure 4A). As each DNA molecule is templated multiple times, but the resulting RNA products are not, polymerase errors are not propagated. Both single-cell ChIP-seq and RNA-seq libraries have been generated using this method (49,190–192). The downside of this approach is that the T7 polymerase is prone to premature termination on low complexity sequences, and if temperatures are reduced to counteract this problem, yield is affected (191). Strand displacement enzymes have been a popular alternative, especially in the context of whole genome amplification (WGA), and to a lesser extent, whole transcriptome amplification. As such, in MDA (multiple strand displacement amplification), DNA is amplified in an isothermal reaction using a random primer and the ϕ29 polymerase (Figure 4B), a very processive enzyme that can generate fragments up to 10 kb from a single template (193). The most efficient templates are either large, linear molecules or circularized molecules (194). As a result, MDA has been successfully applied in various settings, from low-input or single-cell RNA-seq after circularization of cDNA (195,196) to the sequencing of single bacteria in clinical samples (197), or of single tumor cells (198). Despite catalyzing efficient amplification (which is technically not linear), and its high fidelity and very low sequence bias, ∼6% of molecules are chimeras, and amplification bias can still occur due to primer binding skew (199–203). Other strand displacement enzymes used in MDA-type setups include the BstI polymerase and derivatives (204), and a synthetic fusion of the T7 DNA polymerase (3′→5′ exominus) with the processivity-enhancing thioredoxin (marketed as Sequenase), which has successfully been used for low-input ChIP-seq (140) and single-cell RNA-seq (196). In another technique, the MALBAC (multiple annealing and looping-based amplification cycles) method, a strand-displacing enzyme such as BstI is used to generate overlapping fragments from a template using cycles of gradually increasing temperatures and template looping, followed by limited PCR (205) (Figure 4C). The quasilinear amplification step in MALBAC would reportedly result in vastly higher coverage, a lower allele drop-out rate, and a higher reproducibility than MDA for WGA (205,206), although the error rate is lower in MDA due to the higher fidelity of the ϕ29 polymerase (207). Recently, the method has been adapted for single-cell RNA-seq (208).

## NORMALIZATION

Multiple applications benefit from the removal or normalization of abundant nucleic acid sequences, beyond rRNA-derived molecules, in libraries. The large dynamic range of eukaryotic transcriptomes, which spans over four orders of magnitude (209,210), entails that highly expressed transcripts are strongly over-represented in transcriptome libraries. This can be problematic for rare transcript discovery (such as infrequent splicing events) in RNA-seq, and it also needlessly inflates the scale of the library to be screened in approaches relying on RNA as input material but for which transcript abundance information does not need to been retained, such as cDNA expression libraries. Abundant repetitive or organellar sequences in eukaryotic genomes can be a nuisance for some applications, complicating *de novo* genome assembly and alignment (211). Moreover, the sequencing of microbially infected clinical samples (212), of rare (mutated) tumor DNA or RNA in a background of healthy cells, or of fetal cells in a background of abundant maternal cells (213) all represent examples where depletion of unwanted high-abundant RNA or DNA species could substantially increase detection sensitivity.

**Figure 5.**
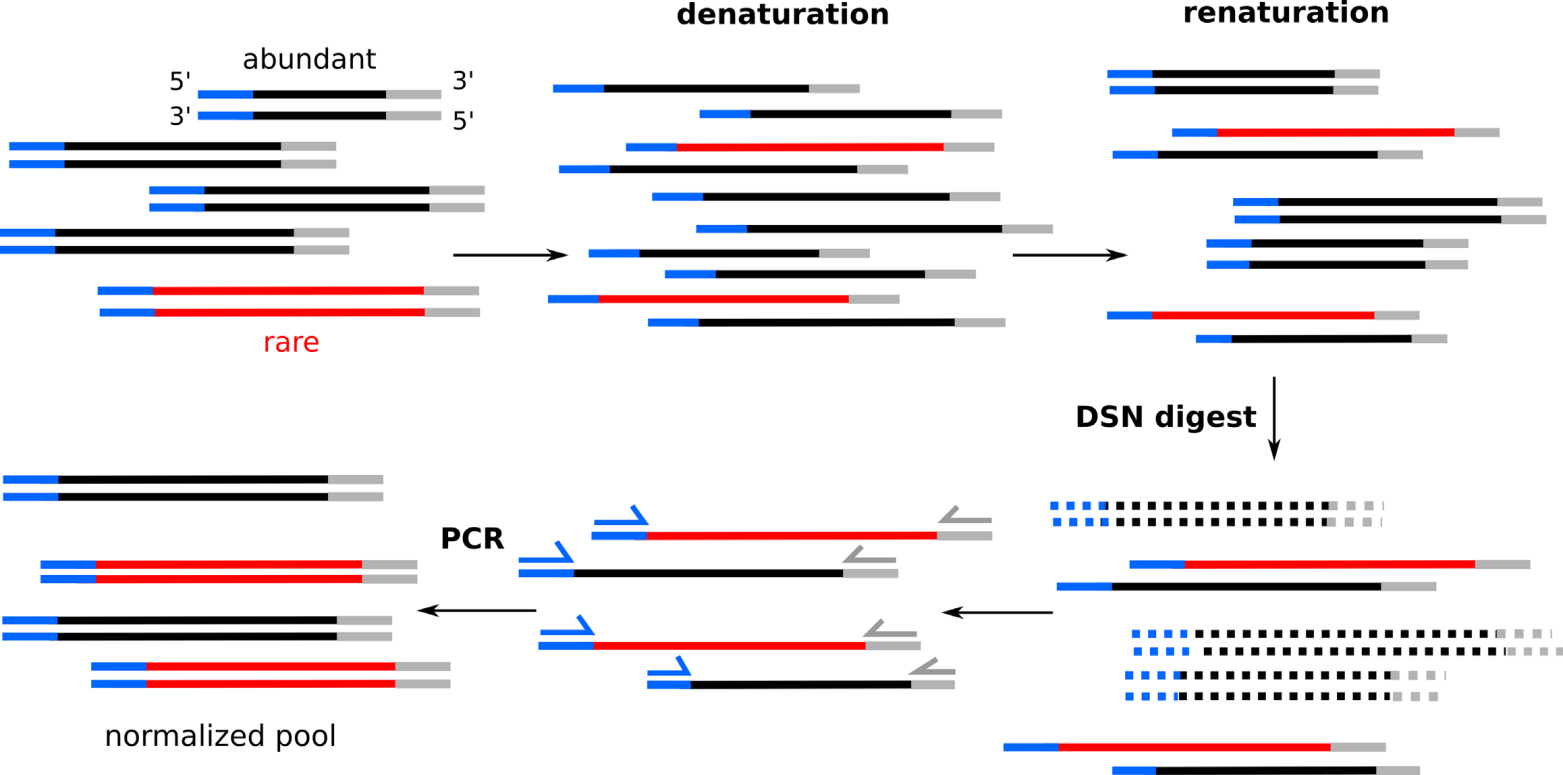
Normalization of DNA abundance with DSN. Adaptor-ligated DNA pools with abundant molecules (black) and rare molecules (red) are subjected to denaturation and controlled slow renaturation at high temperature. Abundant molecules rehybridize faster. This pool of mixed dsDNA and ssDNA is then digested by DSN, which targets duplexes, resulting in unhybridized, single-stranded, low-abundant molecules remaining. A final PCR step enables recovery of these molecules to dsDNA.

Historically, these issues have been addressed in several ways; repetitive sequences, which are often hypermethylated (214,215), have been removed with methylation-specific or methylation-sensitive restriction enzyme systems (216,217), and abundant transcript sequences could be subtracted by hybridization with biotinylated or bead-immobilized driver sequences (218,219). Most often, however, normalization relied on the second-order kinetics of nucleic acid renaturation after denaturation (*DNA concentration ∼ rehybridization rate^2^*); a feature exploited intensely in the context of C_0_t analysis (initial DNA concentration x time) to estimate size, complexity and repetitiveness of genomes before sequencing became the norm (220,221). As abundant DNA sequences reassociate faster than rare ones after denaturation, any method that can reliably separate dsDNA from ssDNA could enrich for low-abundant sequences—most commonly, this was achieved using hydroxyapatite chromatography (222,223). All the above methods proved to be rather labor-intensive (some required substantial skill) and were therefore less suited for higher-throughput studies. The discovery and characterization of a DSN isolated from the hepatopancreas of the Kamchatka crab (*Paralithodes camtschaticus*), however, enabled simple and robust digestion of double-stranded abundant species (224–226) (Figure 5). The DSN enzyme displays a high specificity for DNA in dsDNA or RNA–DNA hybrids of 10 bp or longer, only very little activity on ssDNA, and does not cleave ss or dsRNA, nor does it seem to have any apparent sequence specificity (226,227). As such, it has been efficiently deployed for normalization of cDNA or RNA-seq libraries (224,228–230), reaching up to a 1000-fold reduction in abundance differences (225); but also for genomic DNA normalization (231,232); the removal of specific transcripts (224); and, as mentioned above, ribodepletion (35–37). Additionally, DSN’s ability to discriminate single mismatches in DNA duplexes has successfully been put to use for SNP detection (227). The Michelmore group characterized the global effect of DSN-based normalization through deep sequencing of DNA and RNA libraries, concluding that, for the conditions tested, substantial but not complete abundance equalization was obtained, and that not all sequences seem equally prone to DSN digest (232). Predictably, GC-content plays a role, as high GC% stimulates rehybridization. The addition of TMAC, known to normalize GC and AT pair reannealing rates as exploited in several other applications (183,233–236), could improve this bias and lead to enhanced normalization of AT-rich genes, but it also negatively affected overall normalization efficiency (232). Our own observations suggest that for adaptor-ligated libraries, adaptor sequence can also substantially influence the efficiency of DSN normalization (BioRxiv: http://doi.org/10.1101/241349).

The CRISPR-associated nuclease Cas9 can also been used for similar normalization purposes. DASH could effectively enrich for a rare mutant variant of the *KRAS* gene in synthetic gDNA mixtures with a guide sequence against wild-type *KRAS*, mimicking the situation where rare cancer cells need to be detected in a pool of normal cells (39). This inventive CRISPR-based application can likely easily be extended to remove any combination of sequences of interest from a variety of libraries, as long as good and specific guide RNAs can be designed. Thus, it is anticipated that DASH could complement hybridization-based normalization for sequences that are less efficiently depleted using DSN.

## BARCODES, MOLECULAR TAGS AND FRAMESHIFTS

Despite the high technical reproducibility of next-generation sequencing technologies, batch-to-batch variation effects can still be of concern. Multiplexing samples for sequencing by sample barcoding is a common and recommended approach to reduce part of this variation, while at the same time increasing cost efficiency—provided that the barcodes are well-designed (237). The main culprit for the observed variability between samples, even identical ones, is mostly the multistep library preparation. As such, the earlier samples are barcoded and pooled in the procedure, the better. For single-cell methods, such parallelization provides the additional benefit of increasing total sample amount (238). Shishkin *et al.* recently implemented barcode incorporation during RNA ligation for pooled multiplexed RNA-seq library construction (‘RNAtag-seq’) (239). Similarly, barcodes have been incorporated during cDNA synthesis before pooling (240). Considering the sequence or structural preferences of the various enzymes used during library preparation, it must be noted that exact barcode sequences or their location in the final sequence may also represent a source of bias. miRNA expression profiles, for instance, are known to be significantly skewed when barcodes are introduced adjacent to the ligation site during RNA ligation, but not during PCR amplification (115,241).

Aside from barcoding individual samples, another relatively recent development involves the tagging of individual molecules in single samples through the incorporation of degenerate regions in adaptors or PCR primers before PCR. Such molecular tags (MTs, or unique molecular identifiers, UMIs) have been tremendously useful to differentiate identical molecules originating from the same PCR template (PCR duplicates), and those that were present at the onset of the library preparation (242–246). Sequences with the same UMI can be summarized into consensus sequences, and as such, in applications where the counting of sequences is important, the outcome is less skewed by PCR bias (247) or sequencing errors. UMIs have been successfully applied in the detection of rare variant molecules (248), to accurately profile immune repertoires (249,250) or to quantify mRNA levels from single cells (45,46,48–50), as PCR amplification noise and sequencing errors often obscure these efforts. It has been noted that UMI-based correction does require very high read depths, and that errors in the MTs or barcodes are an issue that should be taken into account (251–253).

**Figure 6.**
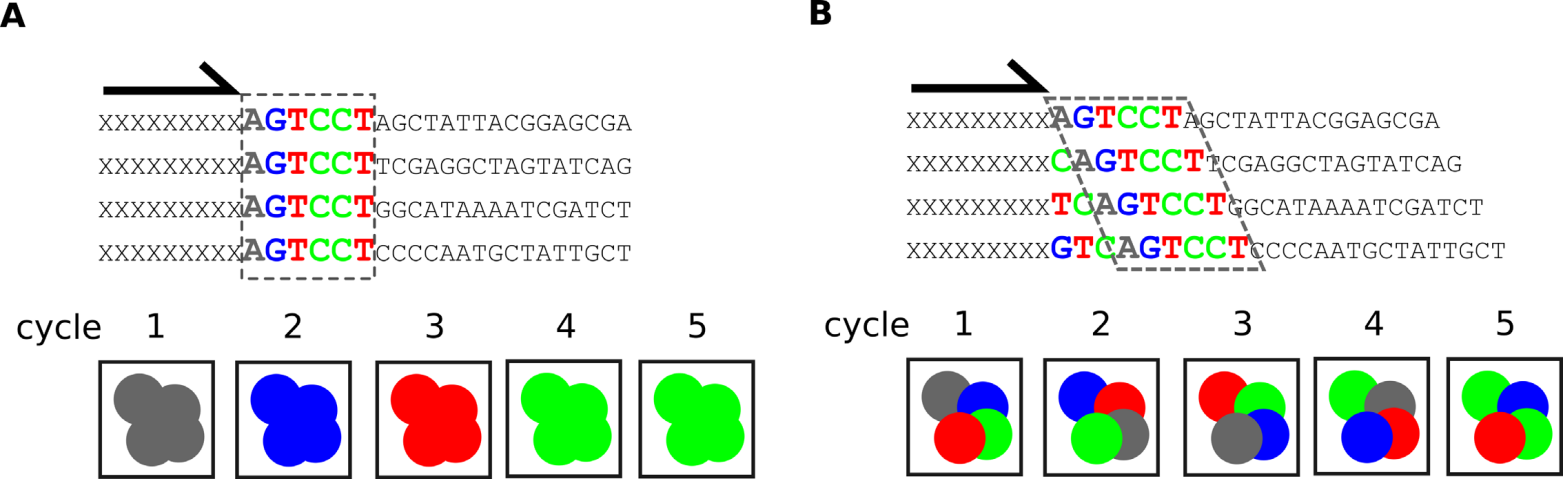
Resolving the issue of low diversity amplicon sequencing on Illumina platforms using frameshifting nucleotides. (**A**) Schematic representation of the sequencing of different molecules with identical starting sequence (e.g. a common primer binding site used for amplification before the addition of Illumina adaptors). Illumina adaptor sequences are represented by Xs. Each molecule symbolizes a sequence cluster on the flow cell. At each cycle, an identical base is read in all clusters, interfering with cluster identification. (**B**) As in A, but here sequences have been amplified with a mix of primers containing additional frameshifting sequences of different lengths. As such, the nucleotide composition at each position in the different clusters is more diverse, enabling more reliable cluster identification. The actual first base of the common region is interrogated at different cycles for each cluster.

As a final note, for libraries of amplicons intended for Illumina sequencing, it may be convenient to introduce sequences of varying lengths just upstream of the first amplicon bases to be sequenced. Illumina platforms strongly rely on the equality of base distributions in the first few cycles for phasing and cluster calling; the sequencing of libraries where the first position is the same in all clusters on the flow cell is therefore very inefficient (254) (Figure 6A). This issue can be bypassed by designing custom sequencing primers (255), but this may require thorough optimization, and is incompatible with paired-end sequencing using older versions of the Illumina control software. Alternatively, mixing in one or more samples with a more random base distribution, such as the PhiX 174 genome, can resolve the problem, but this makes that amplicon samples can never fully benefit from the full chip capacity. Others have reported a custom Illumina sequencing protocol, ‘dark sequencing’, where in a first run clusters are identified in late cycles (after the non-random bases) and the first bases of the sample are sequenced in a second ‘run’ (256). The preferred method, however, involves the incorporation of ‘frameshifting bases’, basically a pool of sequences of varying lengths that are added to the PCR primers. As such, the first sequenced base of each amplicon is different for the different neighbouring clusters (Figure 6B). This strategy has successfully been integrated in several 16S metagenome studies (246,257,258), and ensures full exploitation of the flowcell capacity.

## CONCLUSION

Most molecular manipulations during library preparation introduce some form of bias, resulting in a skewed representation of the original molecules. This can affect accurate quantification, lead to false results, or mask potentially interesting patterns. The nature, source and impact of these library preparation biases in various settings has been subjected to intense research in the past decade, and steadily, strategies to address some of these issues are emerging. As such, TGIRTs and the reverse RTX are showing promise in replacing the inherently more error-prone retroviral RTs, and the benefits of internal randomization of adaptors during RNA ligation have become clear. For DNA amplification, the KAPA HiFi enzyme still tops the charts when it comes to PCR, and with careful PCR cycle number monitoring and the incorporation of MTs, PCR-related data distortions can be attenuated. Linear amplification methods such as MDA and MALBAC are being increasingly used, especially in single-cell setups. The implementation of nucleases such as the DSN or Cas9 for library normalization opens up the prospect of capturing rare molecules in complex samples. These valuable insights should help the researcher to make informed choices when it comes to library generation.

While protocols or enzymes of some commercial kits are generally updated with time, these adaptations often lag behind current knowledge; customizing the library preparation is almost always a better option and generally leads to libraries of superior quality. With continuous effort, it is expected that better enzymes or even simple protocol changes will continue to improve such procedures, enabling more accurate systematic assessment of genome, transcriptome and proteome function.

## References

[1] Heather J.M., Chain B. The sequence of sequencers: the history of sequencing DNA. Genomics. 2016; 107:1–8. 26554401 10.1016/j.ygeno.2015.11.003 PMC4727787

[2] Taipale M., Krykbaeva I., Koeva M., Kayatekin C., Westover K.D., Karras G.I., Lindquist S. Quantitative analysis of HSP90-client interactions reveals principles of substrate recognition. Cell. 2012; 150:987–1001. 22939624 10.1016/j.cell.2012.06.047 PMC3894786

[3] Huttlin E.L., Ting L., Bruckner R.J., Gebreab F., Gygi M.P., Szpyt J., Tam S., Zarraga G., Colby G., Baltier K. The BioPlex Network: a systematic exploration of the human interactome. Cell. 2015; 162:425–440. 26186194 10.1016/j.cell.2015.06.043 PMC4617211

[4] Izhar L., Adamson B., Ciccia A., Lewis J., Pontano-Vaites L., Leng Y., Liang A.C., Westbrook T.F., Harper J.W., Elledge S.J. A systematic analysis of factors localized to damaged chromatin reveals PARP-dependent recruitment of transcription factors. Cell Rep. 2015; 11:1486–1500. 26004182 10.1016/j.celrep.2015.04.053 PMC4464939

[5] Erben E.D., Fadda A., Lueong S., Hoheisel J.D., Clayton C. A genome-wide tethering screen reveals novel potential post-transcriptional regulators in Trypanosoma brucei. PLoS Pathog. 2014; 10:e1004178. 24945722 10.1371/journal.ppat.1004178 PMC4055773

[6] Arnoldo A., Kittanakom S., Heisler L.E., Mak A.B., Shukalyuk A.I., Torti D., Moffat J., Giaever G., Nislow C. A genome scale overexpression screen to reveal drug activity in human cells. Genome Med. 2014; 6:32. 24944581 10.1186/gm549 PMC4062067

[7] The ORFeome Collaboration The ORFeome Collaboration: a genome-scale human ORF-clone resource. Nat. Methods. 2016; 13:191–192. 26914201 10.1038/nmeth.3776

[8] Silva J.M., Li M.Z., Chang K., Ge W., Golding M.C., Rickles R.J., Siolas D., Hu G., Paddison P.J., Schlabach M.R. Second-generation shRNA libraries covering the mouse and human genomes. Nat. Genet. 2005; 37:1281–1288. 16200065 10.1038/ng1650

[9] Horlbeck M.A., Gilbert L.A., Villalta J.E., Adamson B., Pak R.A., Chen Y., Fields A.P., Park C.Y., Corn J.E., Kampmann M. Compact and highly active next-generation libraries for CRISPR-mediated gene repression and activation. eLife. 2016; 5:e19760. 27661255 10.7554/eLife.19760 PMC5094855

[10] Reich S., Puckey L.H., Cheetham C.L., Harris R., Ali A.A.E., Bhattacharyya U., Maclagan K., Powell K.A., Prodromou C., Pearl L.H. Combinatorial Domain Hunting: an effective approach for the identification of soluble protein domains adaptable to high-throughput applications. Protein Sci. Publ. Protein Soc. 2006; 15:2356–2365. 10.1110/ps.062082606 PMC2242398 17008718

[11] Christ D., Winter G. Identification of protein domains by shotgun proteolysis. J. Mol. Biol. 2006; 358:364–371. 16516923 10.1016/j.jmb.2006.01.057

[12] Boxem M., Maliga Z., Klitgord N., Li N., Lemmens I., Mana M., de Lichtervelde L., Mul J.D., van de Peut D., Devos M. A protein domain-based interactome network for C. elegans early embryogenesis. Cell. 2008; 131:534–545. 10.1016/j.cell.2008.07.009 PMC2596478 18692475

[13] Waaijers S., Koorman T., Kerver J., Boxem M. Identification of human protein interaction domains using an ORFeome-based yeast two-hybrid fragment library. J. Proteome Res. 2013; 12:3181–3192. 23718855 10.1021/pr400047p

[14] Linnarsson S. Recent advances in DNA sequencing methods - general principles of sample preparation. Exp. Cell Res. 2010; 316:1339–1343. 20211618 10.1016/j.yexcr.2010.02.036

[15] Head S.R., Komori H.K., Lamere S.A., Whisenant T., Van Nieuwerburgh F., Salomon D.R., Ordoukhanian P. Library construction for next-generation sequencing: overviews and challenges. Biotechniques. 2014; 56:61–77. 24502796 10.2144/000114133 PMC4351865

[16] van Dijk E.L., Jaszczyszyn Y., Thermes C. Library preparation methods for next-generation sequencing: tone down the bias. Exp. Cell Res. 2014; 322:12–20. 24440557 10.1016/j.yexcr.2014.01.008

[17] Ruby J.G., Jan C., Player C., Axtell M.J., Lee W., Nusbaum C., Ge H., Bartel D.P. Large-scale sequencing reveals 21U-RNAs and additional microRNAs and endogenous siRNAs in C. elegans. Cell. 2006; 127:1193–1207. 17174894 10.1016/j.cell.2006.10.040

[18] Lahens N.F., Kavakli I.H., Zhang R., Hayer K., Black M.B., Dueck H., Pizarro A., Kim J., Irizarry R., Thomas R.S. IVT-seq reveals extreme bias in RNA-sequencing. Genome Biol. 2014; 15:R86. 24981968 10.1186/gb-2014-15-6-r86 PMC4197826

[19] Schlackow M., Nojima T., Gomes T., Dhir A., Carmo-Fonseca M., Proudfoot N.J. Distinctive patterns of transcription and RNA processing for human lincRNAs. Mol. Cell. 2017; 65:25–38. 28017589 10.1016/j.molcel.2016.11.029 PMC5222723

[20] Ingolia N.T., Brar G.A., Rouskin S., McGeachy A.M., Weissman J.S. The ribosome profiling strategy for monitoring translation in vivo by deep sequencing of ribosome-protected mRNA fragments. Nat. Protoc. 2012; 7:1534–1550. 22836135 10.1038/nprot.2012.086 PMC3535016

[21] Shen P.S., Park J., Qin Y., Li X., Parsawar K., Larson M.H., Cox J., Cheng Y., Lambowitz A.M., Weissman J.S. Rqc2p and 60S ribosomal subunits mediate mRNA-independent elongation of nascent chains. Science. 2015; 347:75–78. 25554787 10.1126/science.1259724 PMC4451101

[22] Zarnegar B.J., Flynn R.A., Shen Y., Do B.T., Chang H.Y., Khavari P.A. irCLIP platform for efficient characterization of protein-RNA interactions. Nat. Methods. 2016; 13:489–492. 27111506 10.1038/nmeth.3840 PMC5477425

[23] Dai Q., Moshitch-Moshkovitz S., Han D., Kol N., Amariglio N., Rechavi G., Dominissini D., He C. Nm-seq maps 2′-*O*-methylation sites in human mRNA with base precision. Nat. Methods. 2017; 14:695–698. 28504680 10.1038/nmeth.4294 PMC5712428

[24] Rosenow C., Saxena R.M., Durst M., Gingeras T.R. Prokaryotic RNA preparation methods useful for high density array analysis: comparison of two approaches. Nucleic Acids Res. 2001; 29:E112. 11713332 10.1093/nar/29.22.e112 PMC92579

[25] von der Haar T. A quantitative estimation of the global translational activity in logarithmically growing yeast cells. BMC Syst. Biol. 2008; 2:87. 18925958 10.1186/1752-0509-2-87 PMC2590609

[26] Adiconis X., Borges-Rivera D., Satija R., Deluca D.S., Busby M.A., Berlin A.M., Sivachenko A., Thompson D.A., Wysoker A., Fennell T. Comparative analysis of RNA sequencing methods for degraded or low-input samples. Nat. Methods. 2013; 10:623–629. 23685885 10.1038/nmeth.2483 PMC3821180

[27] Sultan M., Amstislavskiy V., Risch T., Schuette M., Dökel S., Ralser M., Balzereit D., Lehrach H., Yaspo M.-L. Influence of RNA extraction methods and library selection schemes on RNA-seq data. BMC Genomics. 2014; 15:675. 25113896 10.1186/1471-2164-15-675 PMC4148917

[28] Dieci G., Fiorino G., Castelnuovo M., Teichmann M., Pagano A. The expanding RNA polymerase III transcriptome. Trends Genet. 2007; 23:614–622. 17977614 10.1016/j.tig.2007.09.001

[29] Yang L., Duff M.O., Graveley B.R., Carmichael G.G., Chen L.-L. Genomewide characterization of non-polyadenylated RNAs. Genome Biol. 2011; 12:R16. 21324177 10.1186/gb-2011-12-2-r16 PMC3188798

[30] Slomovic S., Laufer D., Geiger D., Schuster G. Polyadenylation and degradation of human mitochondrial RNA: the prokaryotic past leaves its mark. Mol. Cell. Biol. 2005; 25:6427–6435. 16024781 10.1128/MCB.25.15.6427-6435.2005 PMC1190340

[31] Nagaike T., Suzuki T., Katoh T., Ueda T. Human mitochondrial mRNAs are stabilized with polyadenylation regulated by mitochondria-specific poly(A) polymerase and polynucleotide phosphorylase. J. Biol. Chem. 2005; 280:19721–19727. 15769737 10.1074/jbc.M500804200

[32] Cui P., Lin Q., Ding F., Xin C., Gong W., Zhang L., Geng J., Zhang B., Yu X., Yang J. A comparison between ribo-minus RNA-sequencing and polyA-selected RNA-sequencing. Genomics. 2010; 96:259–265. 20688152 10.1016/j.ygeno.2010.07.010

[33] Huang R., Jaritz M., Guenzl P., Vlatkovic I., Sommer A., Tamir I.M., Marks H., Klampfl T., Kralovics R., Stunnenberg H.G. An RNA-Seq strategy to detect the complete coding and non-coding transcriptome including full-length imprinted macro ncRNAs. PloS One. 2011; 6:e27288. 22102886 10.1371/journal.pone.0027288 PMC3213133

[34] Weinberg D.E., Shah P., Eichhorn S.W., Hussmann J.A., Plotkin J.B., Bartel D.P. Improved ribosome-footprint and mRNA measurements provide insights into dynamics and regulation of yeast translation. Cell Rep. 2016; 23:1787–1799. 10.1016/j.celrep.2016.01.043 PMC4767672 26876183

[35] Yi H., Cho Y.-J., Won S., Lee J.-E., Jin Yu H., Kim S., Schroth G.P., Luo S., Chun J. Duplex-specific nuclease efficiently removes rRNA for prokaryotic RNA-seq. Nucleic Acids Res. 2011; 39:e140. 21880599 10.1093/nar/gkr617 PMC3203590

[36] Zhao W., He X., Hoadley K.A., Parker J.S., Hayes D.N., Perou C.M. Comparison of RNA-Seq by poly (A) capture, ribosomal RNA depletion, and DNA microarray for expression profiling. BMC Genomics. 2014; 15:419. 24888378 10.1186/1471-2164-15-419 PMC4070569

[37] Fang N., Akinci-Tolun R. Depletion of ribosomal RNA sequences from single-Cell RNA-sequencing library. Curr. Protoc. Mol. Biol. 2016; 115:7.27.1–7.27.20. 10.1002/cpmb.11 27366895

[38] Morlan J.D., Qu K., Sinicropi D.V. Selective depletion of rRNA enables whole transcriptome profiling of archival fixed tissue. PLoS One. 2012; 7:e42882. 22900061 10.1371/journal.pone.0042882 PMC3416766

[39] Gu W., Crawford E.D., O’Donovan B.D., Wilson M.R., Chow E.D., Retallack H., DeRisi J.L. Depletion of abundant sequences by hybridization (DASH): using Cas9 to remove unwanted high-abundance species in sequencing libraries and molecular counting applications. Genome Biol. 2016; 17:41. 26944702 10.1186/s13059-016-0904-5 PMC4778327

[40] Armour C.D., Castle J.C., Chen R., Babak T., Loerch P., Jackson S., Shah J.K., Dey J., Rohl C.A., Johnson J.M. Digital transcriptome profiling using selective hexamer priming for cDNA synthesis. Nat. Methods. 2009; 6:647–649. 19668204 10.1038/nmeth.1360

[41] Arnaud O., Kato S., Poulain S., Plessy C. Targeted reduction of highly abundant transcripts using pseudo-random primers. Biotechniques. 2016; 60:169–174. 27071605 10.2144/000114400

[42] Bhargava V., Ko P., Willems E., Mercola M., Subramaniam S. Quantitative transcriptomics using designed primer-based amplification. Sci. Rep. 2013; 3:1740. 23624976 10.1038/srep01740 PMC3638165

[43] Xu D., Wei G., Lu P., Luo J., Chen X., Skogerbø G., Chen R. Analysis of the p53/CEP-1 regulated non-coding transcriptome in C. elegans by an NSR-seq strategy. Protein Cell. 2014; 5:770–782. 24844773 10.1007/s13238-014-0071-y PMC4180458

[44] Tang F., Barbacioru C., Wang Y., Nordman E., Lee C., Xu N., Wang X., Bodeau J., Tuch B.B., Siddiqui A. mRNA-Seq whole-transcriptome analysis of a single cell. Nat. Methods. 2009; 6:377–382. 19349980 10.1038/nmeth.1315

[45] Hashimshony T., Senderovich N., Avital G., Klochendler A., de Leeuw Y., Anavy L., Gennert D., Li S., Livak K.J., Rozenblatt-Rosen O. CEL-Seq2: sensitive highly-multiplexed single-cell RNA-Seq. Genome Biol. 2016; 17:77. 27121950 10.1186/s13059-016-0938-8 PMC4848782

[46] Fan H.C., Fu G.K., Fodor S.P.A. Expression profiling. Combinatorial labeling of single cells for gene expression cytometry. Science. 2015; 347:1258367. 25657253 10.1126/science.1258367

[47] Picelli S., Björklund Å.K., Faridani O.R., Sagasser S., Winberg G., Sandberg R. Smart-seq2 for sensitive full-length transcriptome profiling in single cells. Nat. Methods. 2013; 10:1096–1098. 24056875 10.1038/nmeth.2639

[48] Islam S., Zeisel A., Joost S., La Manno G., Zajac P., Kasper M., Lönnerberg P., Linnarsson S. Quantitative single-cell RNA-seq with unique molecular identifiers. Nat. Methods. 2014; 11:163–166. 24363023 10.1038/nmeth.2772

[49] Jaitin D.A., Kenigsberg E., Keren-Shaul H., Elefant N., Paul F., Zaretsky I., Mildner A., Cohen N., Jung S., Tanay A. Massively parallel single-cell RNA-seq for marker-free decomposition of tissues into cell types. Science. 2014; 343:776–779. 24531970 10.1126/science.1247651 PMC4412462

[50] Macosko E.Z., Basu A., Satija R., Nemesh J., Shekhar K., Goldman M., Tirosh I., Bialas A.R., Kamitaki N., Martersteck E.M. Highly parallel genome-wide expression profiling of individual cells using nanoliter droplets. Cell. 2015; 161:1202–1214. 26000488 10.1016/j.cell.2015.05.002 PMC4481139

[51] Hayashi T., Ozaki H., Sasagawa Y., Umeda M., Danno H., Nikaido I. Single-cell full-length total RNA sequencing uncovers dynamics of recursive splicing and enhancer RNAs. Nat. Commun. 2018; 9:619. 29434199 10.1038/s41467-018-02866-0 PMC5809388

[52] Mortazavi A., Williams B.A., McCue K., Schaeffer L., Wold B. Mapping and quantifying mammalian transcriptomes by RNA-Seq. Nat. Methods. 2008; 5:621–628. 18516045 10.1038/nmeth.1226 PMC13303166

[53] Breslow R., Huang D.L. Effects of metal ions, including Mg2+ and lanthanides, on the cleavage of ribonucleotides and RNA model compounds. Proc. Natl. Acad. Sci. U.S.A. 1991; 88:4080–4083. 1709734 10.1073/pnas.88.10.4080 PMC51601

[54] Forconi M., Herschlag D. Metal ion-based RNA cleavage as a structural probe. Methods Enzymol. 2009; 468:91–106. 20946766 10.1016/S0076-6879(09)68005-8

[55] Shelton V.M., Morrow J.R. Catalytic transesterification and hydrolysis of RNA by zinc(II) complexes. Inorg. Chem. 1991; 30:4295–4299.

[56] Cameron V., Uhlenbeck O.C. 3′-Phosphatase activity in T4 polynucleotide kinase. Biochemistry (Mosc.). 1977; 16:5120–5126. 10.1021/bi00642a027 199248

[57] Schürer H., Lang K., Schuster J., Mörl M. A universal method to produce in vitro transcripts with homogeneous 3′ ends. Nucleic Acids Res. 2002; 30:e56. 12060694 10.1093/nar/gnf055 PMC117298

[58] Das U., Shuman S. Mechanism of RNA 2′, 3′-cyclic phosphate end healing by T4 polynucleotide kinase-phosphatase. Nucleic Acids Res. 2013; 41:355–365. 23118482 10.1093/nar/gks977 PMC3592404

[59] Ares M. Fragmentation of whole-transcriptome RNA using E. coli RNase III. Cold Spring Harb. Protoc. 2013; 2013:479–481. 23637372 10.1101/pdb.prot074369

[60] MacRae I.J., Doudna J.A. Ribonuclease revisited: structural insights into ribonuclease III family enzymes. Curr. Opin. Struct. Biol. 2007; 17:138–145. 17194582 10.1016/j.sbi.2006.12.002

[61] Wery M., Descrimes M., Thermes C., Gautheret D., Morillon A. Zinc-mediated RNA fragmentation allows robust transcript reassembly upon whole transcriptome RNA-Seq. Methods. 2013; 63:25–31. 23523657 10.1016/j.ymeth.2013.03.009

[62] Yuan Y., Xu H., Leung R.K.-K. An optimized protocol for generation and analysis of Ion Proton sequencing reads for RNA-Seq. BMC Genomics. 2016; 17, doi:10.1186/s12864-016-2745-8. 10.1186/s12864-016-2745-8 PMC4880854 27229683

[63] Min Jou W., Haegeman G., Ysebaert M., Fiers W. Nucleotide sequence of the gene coding for the bacteriophage MS2 coat protein. Nature. 1972; 237:82–88. 4555447 10.1038/237082a0

[64] Ozsolak F., Platt A.R., Jones D.R., Reifenberger J.G., Sass L.E., McInerney P., Thompson J.F., Bowers J., Jarosz M., Milos P.M. Direct RNA sequencing. Nature. 2009; 461:814–818. 19776739 10.1038/nature08390

[65] Garalde D.R., Snell E.A., Jachimowicz D., Sipos B., Lloyd J.H., Bruce M., Pantic N., Admassu T., James P., Warland A. Highly parallel direct RNA sequencing on an array of nanopores. Nat. Methods. 2018; 15:201–206. 29334379 10.1038/nmeth.4577

[66] Xiong Y., Eickbush T.H. Origin and evolution of retroelements based upon their reverse transcriptase sequences. EMBO J. 1990; 9:3353–3362. 1698615 10.1002/j.1460-2075.1990.tb07536.x PMC552073

[67] Arezi B., Hogrefe H. Novel mutations in Moloney Murine Leukemia Virus reverse transcriptase increase thermostability through tighter binding to template-primer. Nucleic Acids Res. 2009; 37:473–481. 19056821 10.1093/nar/gkn952 PMC2632894

[68] Harcourt E.M., Kietrys A.M., Kool E.T. Chemical and structural effects of base modifications in messenger RNA. Nature. 2017; 541:339–346. 28102265 10.1038/nature21351 PMC5498787

[69] Roberts J.D., Bebenek K., Kunkel T.A. The accuracy of reverse transcriptase from HIV-1. Science. 1988; 242:1171–1173. 2460925 10.1126/science.2460925

[70] Menéndez-Arias L. Mutation rates and intrinsic fidelity of retroviral reverse transcriptases. Viruses. 2009; 1:1137–1165. 21994586 10.3390/v1031137 PMC3185545

[71] Ellefson J.W., Gollihar J., Shroff R., Shivram H., Iyer V.R., Ellington A.D. Synthetic evolutionary origin of a proofreading reverse transcriptase. Science. 2016; 352:1590–1593. 27339990 10.1126/science.aaf5409

[72] Hafner M., Renwick N., Brown M., Mihailović A., Holoch D., Lin C., Pena J.T.G., Nusbaum J.D., Morozov P., Ludwig J. RNA-ligase-dependent biases in miRNA representation in deep-sequenced small RNA cDNA libraries. RNA. 2011; 17:1697–1712. 21775473 10.1261/rna.2799511 PMC3162335

[73] Zhuang F., Fuchs R.T., Sun Z., Zheng Y., Robb G.B. Structural bias in T4 RNA ligase-mediated 3′-adapter ligation. Nucleic Acids Res. 2012; 40:e54. 22241775 10.1093/nar/gkr1263 PMC3326334

[74] Haddad F., Qin A.X., Bodell P.W., Zhang L.Y., Guo H., Giger J.M., Baldwin K.M. Regulation of antisense RNA expression during cardiac MHC gene switching in response to pressure overload. Am. J. Physiol. Heart Circ. Physiol. 2006; 290:H2351–H2361. 16415074 10.1152/ajpheart.01111.2005

[75] Haddad F., Qin A.X., Giger J.M., Guo H., Baldwin K.M. Potential pitfalls in the accuracy of analysis of natural sense-antisense RNA pairs by reverse transcription-PCR. BMC Biotechnol. 2007; 7:21. 17480233 10.1186/1472-6750-7-21 PMC1876213

[76] Wu J.Q., Du J., Rozowsky J., Zhang Z., Urban A.E., Euskirchen G., Weissman S., Gerstein M., Snyder M. Systematic analysis of transcribed loci in ENCODE regions using RACE sequencing reveals extensive transcription in the human genome. Genome Biol. 2008; 9:R3. 18173853 10.1186/gb-2008-9-1-r3 PMC2395237

[77] Ruprecht R.M., Goodman N.C., Spiegelman S. Conditions for the selective synthesis of DNA complementary to template RNA. Biochim. Biophys. Acta. 1973; 294:192–203. 4123059

[78] Perocchi F., Xu Z., Clauder-Münster S., Steinmetz L.M. Antisense artifacts in transcriptome microarray experiments are resolved by actinomycin D. Nucleic Acids Res. 2007; 35:e128. 17897965 10.1093/nar/gkm683 PMC2095812

[79] Cocquet J., Chong A., Zhang G., Veitia R.A. Reverse transcriptase template switching and false alternative transcripts. Genomics. 2006; 88:127–131. 16457984 10.1016/j.ygeno.2005.12.013

[80] Roy S.W., Irimia M. When good transcripts go bad: artifactual RT-PCR ‘splicing’ and genome analysis. BioEssays News Rev. Mol. Cell. Dev. Biol. 2008; 30:601–605. 10.1002/bies.20749 18478540

[81] Zajac P., Islam S., Hochgerner H., Lönnerberg P., Linnarsson S. Base preferences in non-templated nucleotide incorporation by MMLV-derived reverse transcriptases. PLoS One. 2013; 8:e85270. 24392002 10.1371/journal.pone.0085270 PMC3877366

[82] Zhu Y.Y., Machleder E.M., Chenchik A., Li R., Siebert P.D. Reverse transcriptase template switching: a SMART approach for full-length cDNA library construction. Biotechniques. 2001; 30:892–897. 11314272 10.2144/01304pf02

[83] Maden B.E., Corbett M.E., Heeney P.A., Pugh K., Ajuh P.M. Classical and novel approaches to the detection and localization of the numerous modified nucleotides in eukaryotic ribosomal RNA. Biochimie. 1995; 77:22–29. 7599273 10.1016/0300-9084(96)88100-4

[84] Munafó D.B., Robb G.B. Optimization of enzymatic reaction conditions for generating representative pools of cDNA from small RNA. RNA. 2010; 16:2537–2552. 20921270 10.1261/rna.2242610 PMC2995414

[85] Kennell J.C., Moran J.V., Perlman P.S., Butow R.A., Lambowitz A.M. Reverse transcriptase activity associated with maturase-encoding group II introns in yeast mitochondria. Cell. 1993; 73:133–146. 7681727 10.1016/0092-8674(93)90166-n

[86] Mohr S., Ghanem E., Smith W., Sheeter D., Qin Y., King O., Polioudakis D., Iyer V.R., Hunicke-Smith S., Swamy S. Thermostable group II intron reverse transcriptase fusion proteins and their use in cDNA synthesis and next-generation RNA sequencing. RNA. 2013; 19:958–970. 23697550 10.1261/rna.039743.113 PMC3683930

[87] Qin Y., Yao J., Wu D.C., Nottingham R.M., Mohr S., Hunicke-Smith S., Lambowitz A.M. High-throughput sequencing of human plasma RNA by using thermostable group II intron reverse transcriptases. RNA. 2015; 22:111–128. 26554030 10.1261/rna.054809.115 PMC4691826

[88] Katibah G.E., Qin Y., Sidote D.J., Yao J., Lambowitz A.M., Collins K. Broad and adaptable RNA structure recognition by the human interferon-induced tetratricopeptide repeat protein IFIT5. Proc. Natl. Acad. Sci. U.S.A. 2014; 111:12025–12030. 25092312 10.1073/pnas.1412842111 PMC4143023

[89] Zheng G., Qin Y., Clark W.C., Dai Q., Yi C., He C., Lambowitz A.M., Pan T. Efficient and quantitative high-throughput tRNA sequencing. Nat. Methods. 2015; 12:835–837. 26214130 10.1038/nmeth.3478 PMC4624326

[90] Nottingham R.M., Wu D.C., Qin Y., Yao J., Hunicke-Smith S., Lambowitz A.M. RNA-seq of human reference RNA samples using a thermostable group II intron reverse transcriptase. RNA. 2016; 22:597–613. 26826130 10.1261/rna.055558.115 PMC4793214

[91] Zhao C., Pyle A.M. Crystal structures of a group II intron maturase reveal a missing link in spliceosome evolution. Nat. Struct. Mol. Biol. 2016; 23:558–565. 27136328 10.1038/nsmb.3224 PMC4899126

[92] Zhao C., Liu F., Pyle A.M. An ultraprocessive, accurate reverse transcriptase encoded by a metazoan group II intron. RNA. 2018; 24:183–195. 29109157 10.1261/rna.063479.117 PMC5769746

[93] Linsen S.E.V., de Wit E., Janssens G., Heater S., Chapman L., Parkin R.K., Fritz B., Wyman S.K., de Bruijn E., Voest E.E. Limitations and possibilities of small RNA digital gene expression profiling. Nat. Methods. 2009; 6:474–476. 19564845 10.1038/nmeth0709-474

[94] Yehudai-Resheff S., Schuster G. Characterization of the E.coli poly(A) polymerase: nucleotide specificity, RNA-binding affinities and RNA structure dependence. Nucleic Acids Res. 2000; 28:1139–1144. 10666455 10.1093/nar/28.5.1139 PMC102612

[95] Raabe C.A., Hoe C.H., Randau G., Brosius J., Tang T.H., Rozhdestvensky T.S. The rocks and shallows of deep RNA sequencing: Examples in the Vibrio cholerae RNome. RNA. 2011; 17:1357–1366. 21610211 10.1261/rna.2682311 PMC3138571

[96] Kirino Y., Mourelatos Z. Mouse Piwi-interacting RNAs are 2′-O-methylated at their 3′ termini. Nat. Struct. Mol. Biol. 2007; 14:347–348. 17384647 10.1038/nsmb1218

[97] Ohara T., Sakaguchi Y., Suzuki T., Ueda H., Miyauchi K., Suzuki T. The 3′ termini of mouse Piwi-interacting RNAs are 2′-O-methylated. Nat. Struct. Mol. Biol. 2007; 14:349–350. 17384646 10.1038/nsmb1220

[98] Raymond C.K., Roberts B.S., Garrett-Engele P., Lim L.P., Johnson J.M. Simple, quantitative primer-extension PCR assay for direct monitoring of microRNAs and short-interfering RNAs. RNA. 2005; 11:1737–1744. 16244135 10.1261/rna.2148705 PMC1370860

[99] Hansen K.D., Brenner S.E., Dudoit S. Biases in Illumina transcriptome sequencing caused by random hexamer priming. Nucleic Acids Res. 2010; 38:e131. 20395217 10.1093/nar/gkq224 PMC2896536

[100] Howland S.W., Poh C.-M., Rénia L. Directional, seamless, and restriction enzyme-free construction of random-primed complementary DNA libraries using phosphorothioate-modified primers. Anal. Biochem. 2011; 416:141–143. 21530478 10.1016/j.ab.2011.04.006

[101] Davis C., Barvish Z., Gitelman I. A method for the construction of equalized directional cDNA libraries from hydrolyzed total RNA. BMC Genomics. 2007; 8:363. 17925018 10.1186/1471-2164-8-363 PMC2134933

[102] Davis C.A., Benzer S. Generation of cDNA expression libraries enriched for in-frame sequences. Proc. Natl. Acad. Sci. U.S.A. 1997; 94:2128–2132. 9122159 10.1073/pnas.94.6.2128 PMC20052

[103] Lyamichev V., Brow M.A., Dahlberg J.E. Structure-specific endonucleolytic cleavage of nucleic acids by eubacterial DNA polymerases. Science. 1993; 260:778–783. 7683443 10.1126/science.7683443

[104] Xu Y., Derbyshire V., Ng K., Sun X.C., Grindley N.D., Joyce C.M. Biochemical and mutational studies of the 5′-3′ exonuclease of DNA polymerase I of Escherichia coli. J. Mol. Biol. 1997; 268:284–302. 9159471 10.1006/jmbi.1997.0967

[105] Fan X., Zhang X., Wu X., Guo H., Hu Y., Tang F., Huang Y. Single-cell RNA-seq transcriptome analysis of linear and circular RNAs in mouse preimplantation embryos. Genome Biol. 2015; 16:148. 26201400 10.1186/s13059-015-0706-1 PMC4511241

[106] Faridani O.R., Abdullayev I., Hagemann-Jensen M., Schell J.P., Lanner F., Sandberg R. Single-cell sequencing of the small-RNA transcriptome. Nat. Biotechnol. 2016; 34:1264–1266. 27798564 10.1038/nbt.3701

[107] Vivancos A.P., Güell M., Dohm J.C., Serrano L., Himmelbauer H. Strand-specific deep sequencing of the transcriptome. Genome Res. 2010; 20:989–999. 20519413 10.1101/gr.094318.109 PMC2892100

[108] Ho C.K., Wang L.K., Lima C.D., Shuman S. Structure and mechanism of RNA ligase. Structure. 2004; 12:327–339. 14962393 10.1016/j.str.2004.01.011

[109] Lau N.C., Lim L.P., Weinstein E.G., Bartel D.P. An abundant class of tiny RNAs with probable regulatory roles in Caenorhabditis elegans. Science. 2001; 294:858–862. 11679671 10.1126/science.1065062

[110] Viollet S., Fuchs R.T., Munafo D.B., Zhuang F., Robb G.B. T4 RNA ligase 2 truncated active site mutants: improved tools for RNA analysis. BMC Biotechnol. 2011; 11:72. 21722378 10.1186/1472-6750-11-72 PMC3149579

[111] Zhelkovsky A.M., McReynolds L.A. Structure-function analysis of Methanobacterium thermoautotrophicum RNA ligase—engineering a thermostable ATP independent enzyme. BMC Mol. Biol. 2012; 13:24. 22809063 10.1186/1471-2199-13-24 PMC3514331

[112] Jackson T.J., Spriggs R.V., Burgoyne N.J., Jones C., Willis A.E. Evaluating bias-reducing protocols for RNA sequencing library preparation. BMC Genomics. 2014; 15:569. 25001197 10.1186/1471-2164-15-569 PMC4117970

[113] Pfeffer S., Sewer A., Lagos-Quintana M., Sheridan R., Sander C., Grässer F.A., van Dyk L.F., Ho C.K., Shuman S., Chien M. Identification of microRNAs of the herpesvirus family. Nat. Methods. 2005; 2:269–276. 15782219 10.1038/nmeth746

[114] Jayaprakash A.D., Jabado O., Brown B.D., Sachidanandam R. Identification and remediation of biases in the activity of RNA ligases in small-RNA deep sequencing. Nucleic Acids Res. 2011; 39:e141. 21890899 10.1093/nar/gkr693 PMC3241666

[115] Alon S., Vigneault F., Eminaga S., Christodoulou D.C., Seidman J.G., Church G.M., Eisenberg E. Barcoding bias in high-throughput multiplex sequencing of miRNA. Genome Res. 2011; 21:1506–1511. 21750102 10.1101/gr.121715.111 PMC3166835

[116] Sorefan K., Pais H., Hall A.E., Kozomara A., Griffiths-Jones S., Moulton V., Dalmay T. Reducing ligation bias of small RNAs in libraries for next generation sequencing. Silence. 2012; 3:4. 22647250 10.1186/1758-907X-3-4 PMC3489589

[117] Sun G., Wu X., Wang J., Li H., Li X., Gao H., Rossi J., Yen Y. A bias-reducing strategy in profiling small RNAs using Solexa. RNA. 2011; 17:2256–2262. 22016383 10.1261/rna.028621.111 PMC3222137

[118] Fuchs R.T., Sun Z., Zhuang F., Robb G.B. Bias in Ligation-Based Small RNA Sequencing Library Construction Is Determined by Adaptor and RNA Structure. PLoS One. 2015; 10:e0126049. 25942392 10.1371/journal.pone.0126049 PMC4420488

[119] Ingolia N.T., Ghaemmaghami S., Newman J.R.S., Weissman J.S. Genome-wide analysis in vivo of translation with nucleotide resolution using ribosome profiling. Science. 2009; 324:218–223. 19213877 10.1126/science.1168978 PMC2746483

[120] Rouskin S., Zubradt M., Washietl S., Kellis M., Weissman J.S. Genome-wide probing of RNA structure reveals active unfolding of mRNA structures in vivo. Nature. 2014; 505:701–705. 24336214 10.1038/nature12894 PMC3966492

[121] Lamm A.T., Stadler M.R., Zhang H., Gent J.I., Fire A.Z. Multimodal RNA-seq using single-strand, double-strand, and CircLigase-based capture yields a refined and extended description of the C. elegans transcriptome. Genome Res. 2011; 21:265–275. 21177965 10.1101/gr.108845.110 PMC3032930

[122] Buermans H.P.J., Ariyurek Y., van Ommen G., den Dunnen J.T., ’t Hoen P.A.C. New methods for next generation sequencing based microRNA expression profiling. BMC Genomics. 2010; 11:716. 21171994 10.1186/1471-2164-11-716 PMC3022920

[123] Zhuang F., Fuchs R.T., Robb G.B. Small RNA expression profiling by high-throughput sequencing: implications of enzymatic manipulation. J. Nucleic Acids. 2012; 2012:360358. 22778911 10.1155/2012/360358 PMC3388297

[124] Raabe C.A., Tang T.-H., Brosius J., Rozhdestvensky T.S. Biases in small RNA deep sequencing data. Nucleic Acids Res. 2014; 42:1414–1426. 24198247 10.1093/nar/gkt1021 PMC3919602

[125] Gubler U., Hoffman B.J. A simple and very efficient method for generating cDNA libraries. Gene. 1983; 25:263–269. 6198242 10.1016/0378-1119(83)90230-5

[126] Parkhomchuk D., Borodina T., Amstislavskiy V., Banaru M., Hallen L., Krobitsch S., Lehrach H., Soldatov A. Transcriptome analysis by strand-specific sequencing of complementary DNA. Nucleic Acids Res. 2009; 37:e123. 19620212 10.1093/nar/gkp596 PMC2764448

[127] Levin J.Z., Yassour M., Adiconis X., Nusbaum C., Thompson D.A., Friedman N., Gnirke A., Regev A. Comprehensive comparative analysis of strand-specific RNA sequencing methods. Nat. Methods. 2010; 7:709–715. 20711195 10.1038/nmeth.1491 PMC3005310

[128] DeGrado-Warren J., Dufford M., Chen J., Bartel P.L., Shattuck D., Frech G.C. Construction and characterization of a normalized yeast two-hybrid library derived from a human protein-coding clone collection. Biotechniques. 2008; 44:265–273. 18330356 10.2144/000112674

[129] Surzycki S. Basic Techniques in Molecular Biology. 2000; Berlin, Heidelberg: Springer.

[130] Quail M.A., Kozarewa I., Smith F., Scally A., Stephens P.J., Durbin R., Swerdlow H., Turner D.J. A large genome center's improvements to the Illumina sequencing system. Nat. Methods. 2008; 5:1005–1010. 19034268 10.1038/nmeth.1270 PMC2610436

[131] Bronner I.F., Quail M.A., Turner D.J., Swerdlow H. Improved Protocols for Illumina Sequencing. Curr. Protoc. Hum. Genet. 2014; 80:18.2.1–18.2.42. 26270174 10.1002/0471142905.hg1802s80

[132] Poptsova M.S., Il’icheva I.A., Nechipurenko D.Y., Panchenko L.A., Khodikov M.V., Oparina N.Y., Polozov R.V., Nechipurenko Y.D., Grokhovsky S.L. Non-random DNA fragmentation in next-generation sequencing. Sci. Rep. 2014; 4:4532. 24681819 10.1038/srep04532 PMC3970190

[133] Crawford G.E., Davis S., Scacheri P.C., Renaud G., Halawi M.J., Erdos M.R., Green R., Meltzer P.S., Wolfsberg T.G., Collins F.S. DNase-chip: a high-resolution method to identify DNase I hypersensitive sites using tiled microarrays. Nat. Methods. 2006; 3:503–509. 16791207 10.1038/NMETH888 PMC2698431

[134] Sabo P.J., Kuehn M.S., Thurman R., Johnson B.E., Johnson E.M., Cao H., Yu M., Rosenzweig E., Goldy J., Haydock A. Genome-scale mapping of DNase I sensitivity in vivo using tiling DNA microarrays. Nat. Methods. 2006; 3:511–518. 16791208 10.1038/nmeth890

[135] Koohy H., Down T.A., Hubbard T.J. Chromatin accessibility data sets show bias due to sequence specificity of the DNase I enzyme. PLoS One. 2013; 8:e69853. 23922824 10.1371/journal.pone.0069853 PMC3724795

[136] Aigrain L., Gu Y., Quail M.A. Quantitation of next generation sequencing library preparation protocol efficiencies using droplet digital PCR assays - a systematic comparison of DNA library preparation kits for Illumina sequencing. BMC Genomics. 2016; 17:458. 27297323 10.1186/s12864-016-2757-4 PMC4906846

[137] Knierim E., Lucke B., Schwarz J.M., Schuelke M., Seelow D. Systematic comparison of three methods for fragmentation of long-range PCR products for next generation sequencing. PLoS One. 2011; 6:e28240. 22140562 10.1371/journal.pone.0028240 PMC3227650

[138] Grothues D., Cantor C.R., Smith C.L. PCR amplification of megabase DNA with tagged random primers (T-PCR). Nucleic Acids Res. 1993; 21:1321–1322. 8464718 10.1093/nar/21.5.1321 PMC309304

[139] Kawasaki M., Inagaki F. Random PCR-based screening for soluble domains using green fluorescent protein. Biochem. Biophys. Res. Commun. 2001; 280:842–844. 11162598 10.1006/bbrc.2000.4229

[140] Adli M., Zhu J., Bernstein B.E. Genome-wide chromatin maps derived from limited numbers of hematopoietic progenitors. Nat. Methods. 2010; 7:615–618. 20622861 10.1038/nmeth.1478 PMC2924612

[141] Prodromou C., Savva R., Driscoll P.C. DNA fragmentation-based combinatorial approaches to soluble protein expression Part I. Generating DNA fragment libraries. Drug Discov. Today. 2007; 12:931–938. 17993411 10.1016/j.drudis.2007.08.012

[142] Maclagan K., Tommasi R., Laurine E., Prodromou C., Driscoll P.C., Pearl L.H., Reich S., Savva R. A combinatorial method to enable detailed investigation of protein-protein interactions. Future Med. Chem. 2011; 3:271–282. 21446842 10.4155/fmc.10.289

[143] Miyazaki K. Random DNA fragmentation with endonuclease V: application to DNA shuffling. Nucleic Acids Res. 2002; 30:e139. 12490730 10.1093/nar/gnf139 PMC140088

[144] Dyson M.R., Perera R.L., Shadbolt S.P., Biderman L., Bromek K., Murzina N.V., McCafferty J. Identification of soluble protein fragments by gene fragmentation and genetic selection. Nucleic Acids Res. 2008; 36:e51. 18420658 10.1093/nar/gkn151 PMC2396403

[145] Wang K., Koop B.F., Hood L. A simple method using T4 DNA polymerase to clone polymerase chain reaction products. Biotechniques. 1994; 17:236–238. 7980913

[146] Zheng Z., Advani A., Melefors O., Glavas S., Nordström H., Ye W., Engstrand L., Andersson A.F. Titration-free 454 sequencing using Y adapters. Nat. Protoc. 2011; 6:1367–1376. 21886102 10.1038/nprot.2011.369

[147] Jain M., Fiddes I.T., Miga K.H., Olsen H.E., Paten B., Akeson M. Improved data analysis for the MinION nanopore sequencer. Nat. Methods. 2015; 12:351–356. 25686389 10.1038/nmeth.3290 PMC4907500

[148] Agarwal S., Macfarlan T.S., Sartor M.A., Iwase S. Sequencing of first-strand cDNA library reveals full-length transcriptomes. Nat. Commun. 2015; 6:6002. 25607527 10.1038/ncomms7002 PMC5054741

[149] Makarov V., Laliberte J. Swift Biosciences, I. Improved methods for processing DNA substrates. 2015; Patent CA2938213 A1.

[150] Gorbacheva T., Quispe-Tintaya W., Popov V.N., Vijg J., Maslov A.Y. Improved transposon-based library preparation for the Ion Torrent platform. Biotechniques. 2015; 58:200–202. 25861933 10.2144/000114277 PMC5494161

[151] Adey A., Morrison H.G., Asan, Xun X., Kitzman J.O., Turner E.H., Stackhouse B., MacKenzie A.P., Caruccio N.C., Zhang X. Rapid, low-input, low-bias construction of shotgun fragment libraries by high-density in vitro transposition. Genome Biol. 2010; 11:R119. 21143862 10.1186/gb-2010-11-12-r119 PMC3046479

[152] Lan J.H., Yin Y., Reed E.F., Moua K., Thomas K., Zhang Q. Impact of three Illumina library construction methods on GC bias and HLA genotype calling. Hum. Immunol. 2015; 76:166–175. 25543015 10.1016/j.humimm.2014.12.016 PMC5089167

[153] Tin M.M.-Y., Economo E.P., Mikheyev A.S. Sequencing degraded DNA from non-destructively sampled museum specimens for RAD-tagging and low-coverage shotgun phylogenetics. PLoS One. 2014; 9:e96793. 24828244 10.1371/journal.pone.0096793 PMC4020769

[154] Turchinovich A., Surowy H., Serva A., Zapatka M., Lichter P., Burwinkel B. Capture and amplification by tailing and switching (CATS). an ultrasensitive ligation-independent method for generation of DNA libraries for deep sequencing from picogram amounts of DNA and RNA. RNA Biol. 2014; 11:817–828. 24922482 10.4161/rna.29304 PMC4179956

[155] Gansauge M.-T., Meyer M. Single-stranded DNA library preparation for the sequencing of ancient or damaged DNA. Nat. Protoc. 2013; 8:737–748. 23493070 10.1038/nprot.2013.038

[156] Gansauge M.-T., Gerber T., Glocke I., Korlevic P., Lippik L., Nagel S., Riehl L.M., Schmidt A., Meyer M. Single-stranded DNA library preparation from highly degraded DNA using T4 DNA ligase. Nucleic Acids Res. 2017; 45:e79. 28119419 10.1093/nar/gkx033 PMC5449542

[157] Plongthongkum N., Diep D.H., Zhang K. Advances in the profiling of DNA modifications: cytosine methylation and beyond. Nat. Rev. Genet. 2014; 15:647–661. 25159599 10.1038/nrg3772

[158] Frommer M., McDonald L.E., Millar D.S., Collis C.M., Watt F., Grigg G.W., Molloy P.L., Paul C.L. A genomic sequencing protocol that yields a positive display of 5-methylcytosine residues in individual DNA strands. Proc. Natl. Acad. Sci. U.S.A. 1992; 89:1827–1831. 1542678 10.1073/pnas.89.5.1827 PMC48546

[159] Tanaka K., Okamoto A. Degradation of DNA by bisulfite treatment. Bioorg. Med. Chem. Lett. 2007; 17:1912–1915. 17276678 10.1016/j.bmcl.2007.01.040

[160] Lister R., O’Malley R.C., Tonti-Filippini J., Gregory B.D., Berry C.C., Millar A.H., Ecker J.R. Highly integrated single-base resolution maps of the epigenome in Arabidopsis. Cell. 2008; 133:523–536. 18423832 10.1016/j.cell.2008.03.029 PMC2723732

[161] Cokus S.J., Feng S., Zhang X., Chen Z., Merriman B., Haudenschild C.D., Pradhan S., Nelson S.F., Pellegrini M., Jacobsen S.E. Shotgun bisulphite sequencing of the Arabidopsis genome reveals DNA methylation patterning. Nature. 2008; 452:215–219. 18278030 10.1038/nature06745 PMC2377394

[162] Miura F., Enomoto Y., Dairiki R., Ito T. Amplification-free whole-genome bisulfite sequencing by post-bisulfite adaptor tagging. Nucleic Acids Res. 2012; 40:e136. 22649061 10.1093/nar/gks454 PMC3458524

[163] Farlik M., Sheffield N.C., Nuzzo A., Datlinger P., Schönegger A., Klughammer J., Bock C. Single-cell DNA methylome sequencing and bioinformatic inference of epigenomic cell-state dynamics. Cell Rep. 2015; 10:1386–1397. 25732828 10.1016/j.celrep.2015.02.001 PMC4542311

[164] Smallwood S.A., Lee H.J., Angermueller C., Krueger F., Saadeh H., Peat J., Andrews S.R., Stegle O., Reik W., Kelsey G. Single-cell genome-wide bisulfite sequencing for assessing epigenetic heterogeneity. Nat. Methods. 2014; 11:817–820. 25042786 10.1038/nmeth.3035 PMC4117646

[165] Raine A., Manlig E., Wahlberg P., Syvänen A.-C., Nordlund J. SPlinted Ligation Adapter Tagging (SPLAT), a novel library preparation method for whole genome bisulphite sequencing. Nucleic Acids Res. 2017; 45:e36. 27899585 10.1093/nar/gkw1110 PMC5389478

[166] Meissner A., Gnirke A., Bell G.W., Ramsahoye B., Lander E.S., Jaenisch R. Reduced representation bisulfite sequencing for comparative high-resolution DNA methylation analysis. Nucleic Acids Res. 2005; 33:5868–5877. 16224102 10.1093/nar/gki901 PMC1258174

[167] Meissner A., Mikkelsen T.S., Gu H., Wernig M., Hanna J., Sivachenko A., Zhang X., Bernstein B.E., Nusbaum C., Jaffe D.B. Genome-scale DNA methylation maps of pluripotent and differentiated cells. Nature. 2008; 454:766–770. 18600261 10.1038/nature07107 PMC2896277

[168] Martin-Herranz D.E., Ribeiro A.J.M., Krueger F., Thornton J.M., Reik W., Stubbs T.M. cuRRBS: simple and robust evaluation of enzyme combinations for reduced representation approaches. Nucleic Acids Res. 2017; 45:11559–11569. 29036576 10.1093/nar/gkx814 PMC5714207

[169] Guo H., Zhu P., Guo F., Li X., Wu X., Fan X., Wen L., Tang F. Profiling DNA methylome landscapes of mammalian cells with single-cell reduced-representation bisulfite sequencing. Nat. Protoc. 2015; 10:645–659. 25837417 10.1038/nprot.2015.039

[170] Wen L., Li J., Guo H., Liu X., Zheng S., Zhang D., Zhu W., Qu J., Guo L., Du D. Genome-scale detection of hypermethylated CpG islands in circulating cell-free DNA of hepatocellular carcinoma patients. Cell Res. 2015; 25:1250–1264. 26516143 10.1038/cr.2015.126 PMC4650428

[171] Tanić M., Beck S. Epigenome-wide association studies for cancer biomarker discovery in circulating cell-free DNA: technical advances and challenges. Curr. Opin. Genet. Dev. 2017; 42:48–55. 28391083 10.1016/j.gde.2017.01.017

[172] Serre D., Lee B.H., Ting A.H. MBD-isolated Genome Sequencing provides a high-throughput and comprehensive survey of DNA methylation in the human genome. Nucleic Acids Res. 2010; 38:391–399. 19906696 10.1093/nar/gkp992 PMC2811030

[173] Brinkman A.B., Simmer F., Ma K., Kaan A., Zhu J., Stunnenberg H.G. Whole-genome DNA methylation profiling using MethylCap-seq. Methods. 2010; 52:232–236. 20542119 10.1016/j.ymeth.2010.06.012

[174] Down T.A., Rakyan V.K., Turner D.J., Flicek P., Li H., Kulesha E., Gräf S., Johnson N., Herrero J., Tomazou E.M. A Bayesian deconvolution strategy for immunoprecipitation-based DNA methylome analysis. Nat. Biotechnol. 2008; 26:779–785. 18612301 10.1038/nbt1414 PMC2644410

[175] Kebschull J.M., Zador A.M. Sources of PCR-induced distortions in high-throughput sequencing data sets. Nucleic Acids Res. 2015; 43:e143. 26187991 10.1093/nar/gkv717 PMC4666380

[176] D’Amore R., Ijaz U.Z., Schirmer M., Kenny J.G., Gregory R., Darby A.C., Shakya M., Podar M., Quince C., Hall N. A comprehensive benchmarking study of protocols and sequencing platforms for 16S rRNA community profiling. BMC Genomics. 2016; 17:55. 26763898 10.1186/s12864-015-2194-9 PMC4712552

[177] Polz M.F., Cavanaugh C.M. Bias in template-to-product ratios in multitemplate PCR. Appl. Environ. Microbiol. 1998; 64:3724–3730. 9758791 10.1128/aem.64.10.3724-3730.1998 PMC106531

[178] Qiu X., Wu L., Huang H., McDonel P.E., Palumbo A.V., Tiedje J.M., Zhou J. Evaluation of PCR-generated chimeras, mutations, and heteroduplexes with 16S rRNA gene-based cloning. Appl. Environ. Microbiol. 2001; 67:880–887. 11157258 10.1128/AEM.67.2.880-887.2001 PMC92662

[179] Ahn J.-H., Kim B.-Y., Song J., Weon H.-Y. Effects of PCR cycle number and DNA polymerase type on the 16S rRNA gene pyrosequencing analysis of bacterial communities. J. Microbiol. 2012; 50:1071–1074. 23274999 10.1007/s12275-012-2642-z

[180] Dabney J., Meyer M. Length and GC-biases during sequencing library amplification: a comparison of various polymerase-buffer systems with ancient and modern DNA sequencing libraries. Biotechniques. 2012; 52:87–94. 22313406 10.2144/000113809

[181] Williams R., Peisajovich S.G., Miller O.J., Magdassi S., Tawfik D.S., Griffiths A.D. Amplification of complex gene libraries by emulsion PCR. Nat. Methods. 2006; 3:545–550. 16791213 10.1038/nmeth896

[182] Aird D., Ross M.G., Chen W.-S., Danielsson M., Fennell T., Russ C., Jaffe D.B., Nusbaum C., Gnirke A. Analyzing and minimizing PCR amplification bias in Illumina sequencing libraries. Genome Biol. 2011; 12:R18. 21338519 10.1186/gb-2011-12-2-r18 PMC3188800

[183] Oyola S.O., Otto T.D., Gu Y., Maslen G., Manske M., Campino S., Turner D.J., Macinnis B., Kwiatkowski D.P., Swerdlow H.P. Optimizing Illumina next-generation sequencing library preparation for extremely AT-biased genomes. BMC Genomics. 2012; 13:1. 22214261 10.1186/1471-2164-13-1 PMC3312816

[184] Quail M.A., Otto T.D., Gu Y., Harris S.R., Skelly T.F., McQuillan J.A., Swerdlow H.P., Oyola S.O. Optimal enzymes for amplifying sequencing libraries. Nat. Meth. 2012; 9:10–11. 10.1038/nmeth.1814 22205512

[185] Gohl D.M., Vangay P., Garbe J., MacLean A., Hauge A., Becker A., Gould T.J., Clayton J.B., Johnson T.J., Hunter R. Systematic improvement of amplicon marker gene methods for increased accuracy in microbiome studies. Nat. Biotechnol. 2016; 9:942–949. 10.1038/nbt.3601 27454739

[186] Kozarewa I., Ning Z., Quail M.A., Sanders M.J., Berriman M., Turner D.J. Amplification-free Illumina sequencing-library preparation facilitates improved mapping and assembly of (G+C)-biased genomes. Nat. Methods. 2009; 6:291–295. 19287394 10.1038/nmeth.1311 PMC2664327

[187] Mamanova L., Coffey A.J., Scott C.E., Kozarewa I., Turner E.H., Kumar A., Howard E., Shendure J., Turner D.J. Target-enrichment strategies for next-generation sequencing. Nat. Methods. 2010; 7:111–118. 20111037 10.1038/nmeth.1419

[188] Hamatani T., Carter M.G., Sharov A.A., Ko M.S.H. Dynamics of global gene expression changes during mouse preimplantation development. Dev. Cell. 2004; 6:117–131. 14723852 10.1016/s1534-5807(03)00373-3

[189] Schneider J., Buness A., Huber W., Volz J., Kioschis P., Hafner M., Poustka A., Sültmann H. Systematic analysis of T7 RNA polymerase based in vitro linear RNA amplification for use in microarray experiments. BMC Genomics. 2004; 5:29. 15119961 10.1186/1471-2164-5-29 PMC419340

[190] Bártfai R., Hoeijmakers W.A.M., Salcedo-Amaya A.M., Smits A.H., Janssen-Megens E., Kaan A., Treeck M., Gilberger T.-W., Françoijs K.-J., Stunnenberg H.G. H2A.Z Demarcates Intergenic Regions of the Plasmodium falciparum Epigenome That Are Dynamically Marked by H3K9ac and H3K4me3. PLoS Pathog. 2010; 6:e1001223. 21187892 10.1371/journal.ppat.1001223 PMC3002978

[191] Hoeijmakers W.A.M., Bartfai R., Francoijs K.-J., Stunnenberg H.G. Linear amplification for deep sequencing. Nat. Protoc. 2011; 6:1026–1036. 21720315 10.1038/nprot.2011.345

[192] Hashimshony T., Wagner F., Sher N., Yanai I. CEL-Seq: single-cell RNA-Seq by multiplexed linear amplification. Cell Rep. 2012; 2:666–673. 22939981 10.1016/j.celrep.2012.08.003

[193] Dean F.B., Nelson J.R., Giesler T.L., Lasken R.S. Rapid amplification of plasmid and phage DNA using Phi 29 DNA polymerase and multiply-primed rolling circle amplification. Genome Res. 2001; 11:1095–1099. 11381035 10.1101/gr.180501 PMC311129

[194] Shoaib M., Baconnais S., Mechold U., Le Cam E., Lipinski M., Ogryzko V. Multiple displacement amplification for complex mixtures of DNA fragments. BMC Genomics. 2008; 9:415. 18793430 10.1186/1471-2164-9-415 PMC2553422

[195] Pan X., Urban A.E., Palejev D., Schulz V., Grubert F., Hu Y., Snyder M., Weissman S.M. A procedure for highly specific, sensitive, and unbiased whole-genome amplification. Proc. Natl. Acad. Sci. U.S.A. 2008; 105:15499–15504. 18832167 10.1073/pnas.0808028105 PMC2563063

[196] Pan X., Durrett R.E., Zhu H., Tanaka Y., Li Y., Zi X., Marjani S.L., Euskirchen G., Ma C., Lamotte R.H. Two methods for full-length RNA sequencing for low quantities of cells and single cells. Proc. Natl. Acad. Sci. U.S.A. 2013; 110:594–599. 23267071 10.1073/pnas.1217322109 PMC3545756

[197] Seth-Smith H.M.B., Harris S.R., Scott P., Parmar S., Marsh P., Unemo M., Clarke I.N., Parkhill J., Thomson N.R. Generating whole bacterial genome sequences of low-abundance species from complex samples with IMS-MDA. Nat. Protoc. 2013; 8:2404–2412. 24202554 10.1038/nprot.2013.147

[198] Navin N., Kendall J., Troge J., Andrews P., Rodgers L., McIndoo J., Cook K., Stepansky A., Levy D., Esposito D. Tumour evolution inferred by single-cell sequencing. Nature. 2011; 472:90–94. 21399628 10.1038/nature09807 PMC4504184

[199] Paez J.G., Lin M., Beroukhim R., Lee J.C., Zhao X., Richter D.J., Gabriel S., Herman P., Sasaki H., Altshuler D. Genome coverage and sequence fidelity of phi29 polymerase-based multiple strand displacement whole genome amplification. Nucleic Acids Res. 2004; 32:e71. 15150323 10.1093/nar/gnh069 PMC419624

[200] Zhang K., Martiny A.C., Reppas N.B., Barry K.W., Malek J., Chisholm S.W., Church G.M. Sequencing genomes from single cells by polymerase cloning. Nat. Biotechnol. 2006; 24:680–686. 16732271 10.1038/nbt1214

[201] Chitsaz H., Yee-Greenbaum J.L., Tesler G., Lombardo M.-J., Dupont C.L., Badger J.H., Novotny M., Rusch D.B., Fraser L.J., Gormley N.A. Efficient de novo assembly of single-cell bacterial genomes from short-read data sets. Nat. Biotechnol. 2011; 29:915–921. 21926975 10.1038/nbt.1966 PMC3558281

[202] Hasmats J., Gréen H., Orear C., Validire P., Huss M., Käller M., Lundeberg J. Assessment of whole genome amplification for sequence capture and massively parallel sequencing. PLoS One. 2014; 9:e84785. 24409309 10.1371/journal.pone.0084785 PMC3883664

[203] Tu J., Guo J., Li J., Gao S., Yao B., Lu Z. Systematic characteristic exploration of the chimeras generated in multiple displacement amplification through next generation sequencing data reanalysis. PLoS One. 2015; 10:e0139857. 26440104 10.1371/journal.pone.0139857 PMC4595205

[204] Lage J.M., Leamon J.H., Pejovic T., Hamann S., Lacey M., Dillon D., Segraves R., Vossbrinck B., González A., Pinkel D. Whole genome analysis of genetic alterations in small DNA samples using hyperbranched strand displacement amplification and array-CGH. Genome Res. 2003; 13:294–307. 12566408 10.1101/gr.377203 PMC420367

[205] Zong C., Lu S., Chapman A.R., Xie X.S. Genome-wide detection of single-nucleotide and copy-number variations of a single human cell. Science. 2012; 338:1622–1626. 23258894 10.1126/science.1229164 PMC3600412

[206] Chen M., Song P., Zou D., Hu X., Zhao S., Gao S., Ling F. Comparison of multiple displacement amplification (MDA) and multiple annealing and looping-based amplification cycles (MALBAC) in single-cell sequencing. PLoS One. 2014; 9:e114520. 25485707 10.1371/journal.pone.0114520 PMC4259343

[207] de Bourcy C.F.A., De Vlaminck I., Kanbar J.N., Wang J., Gawad C., Quake S.R. A quantitative comparison of single-cell whole genome amplification methods. PLoS One. 2014; 9:e105585. 25136831 10.1371/journal.pone.0105585 PMC4138190

[208] Chapman A.R., He Z., Lu S., Yong J., Tan L., Tang F., Xie X.S. Single cell transcriptome amplification with MALBAC. PLoS One. 2015; 10:e0120889. 25822772 10.1371/journal.pone.0120889 PMC4378937

[209] Vogel C., de Sousa Abreu R., Ko D., Le S.-Y., Shapiro B.A., Burns S.C., Sandhu D., Boutz D.R., Marcotte E.M., Penalva L.O. Sequence signatures and mRNA concentration can explain two-thirds of protein abundance variation in a human cell line. Mol. Syst. Biol. 2010; 6:400. 20739923 10.1038/msb.2010.59 PMC2947365

[210] Schwanhäusser B., Busse D., Li N., Dittmar G., Schuchhardt J., Wolf J., Chen W., Selbach M. Global quantification of mammalian gene expression control. Nature. 2011; 473:337–342. 21593866 10.1038/nature10098

[211] De Schutter K., Lin Y.-C., Tiels P., Van Hecke A., Glinka S., Weber-Lehmann J., Rouzé P., Van de Peer Y., Callewaert N. Genome sequence of the recombinant protein production host Pichia pastoris. Nat. Biotechnol. 2009; 27:561–566. 19465926 10.1038/nbt.1544

[212] Bukowska-Ośko I., Perlejewski K., Nakamura S., Motooka D., Stokowy T., Kosińska J., Popiel M., Płoski R., Horban A., Lipowski D. Sensitivity of next-generation sequencing metagenomic analysis for detection of RNA and DNA viruses in cerebrospinal fluid: the confounding effect of background contamination. Adv. Exp. Med. Biol. 2016; 944:53–62. 10.1007/5584_2016_42 27405447

[213] Fan H.C., Gu W., Wang J., Blumenfeld Y.J., El-Sayed Y.Y., Quake S.R. Non-invasive prenatal measurement of the fetal genome. Nature. 2012; 487:320–324. 22763444 10.1038/nature11251 PMC3561905

[214] Hata K., Sakaki Y. Identification of critical CpG sites for repression of L1 transcription by DNA methylation. Gene. 1997; 189:227–234. 9168132 10.1016/s0378-1119(96)00856-6

[215] Su J., Shao X., Liu H., Liu S., Wu Q., Zhang Y. Genome-wide dynamic changes of DNA methylation of repetitive elements in human embryonic stem cells and fetal fibroblasts. Genomics. 2012; 99:10–17. 22044633 10.1016/j.ygeno.2011.10.004

[216] Rabinowicz P.D., Schutz K., Dedhia N., Yordan C., Parnell L.D., Stein L., McCombie W.R., Martienssen R.A. Differential methylation of genes and retrotransposons facilitates shotgun sequencing of the maize genome. Nat. Genet. 1999; 23:305–308. 10545948 10.1038/15479

[217] Emberton J., Ma J., Yuan Y., SanMiguel P., Bennetzen J.L. Gene enrichment in maize with hypomethylated partial restriction (HMPR) libraries. Genome Res. 2005; 15:1441–1446. 16204197 10.1101/gr.3362105 PMC1240087

[218] Sasaki Y.F., Ayusawa D., Oishi M. Construction of a normalized cDNA library by introduction of a semi-solid mRNA-cDNA hybridization system. Nucleic Acids Res. 1994; 22:987–992. 8152931 10.1093/nar/22.6.987 PMC307919

[219] Carninci P., Shibata Y., Hayatsu N., Sugahara Y., Shibata K., Itoh M., Konno H., Okazaki Y., Muramatsu M., Hayashizaki Y. Normalization and subtraction of cap-trapper-selected cDNAs to prepare full-length cDNA libraries for rapid discovery of new genes. Genome Res. 2000; 10:1617–1630. 11042159 10.1101/gr.145100 PMC310980

[220] Peterson D.G., Schulze S.R., Sciara E.B., Lee S.A., Bowers J.E., Nagel A., Jiang N., Tibbitts D.C., Wessler S.R., Paterson A.H. Integration of cot analysis, DNA cloning, and high-throughput sequencing facilitates genome characterization and gene discovery. Genome Res. 2002; 12:795–807. 11997346 10.1101/gr.226102 PMC186575

[221] Paterson A.H. Leafing through the genomes of our major crop plants: strategies for capturing unique information. Nat. Rev. Genet. 2006; 7:174–184. 16485017 10.1038/nrg1806

[222] Patanjali S.R., Parimoo S., Weissman S.M. Construction of a uniform-abundance (normalized) cDNA library. Proc. Natl. Acad. Sci. U.S.A. 1991; 88:1943–1947. 1705712 10.1073/pnas.88.5.1943 PMC51142

[223] Vandernoot V.A., Langevin S.A., Solberg O.D., Lane P.D., Curtis D.J., Bent Z.W., Williams K.P., Patel K.D., Schoeniger J.S., Branda S.S. cDNA normalization by hydroxyapatite chromatography to enrich transcriptome diversity in RNA-seq applications. Biotechniques. 2012; 53:373–380. 23227988 10.2144/000113937

[224] Zhulidov P.A., Bogdanova E.A., Shcheglov A.S., Vagner L.L., Khaspekov G.L., Kozhemyako V.B., Matz M.V., Meleshkevitch E., Moroz L.L., Lukyanov S.A. Simple cDNA normalization using kamchatka crab duplex-specific nuclease. Nucleic Acids Res. 2004; 32:e37. 14973331 10.1093/nar/gnh031 PMC373426

[225] Anisimova V.E., Rebrikov D.V., Zhulidov P.A., Staroverov D.B., Lukyanov S.A., Shcheglov A.S. Renaturation, activation, and practical use of recombinant duplex-specific nuclease from Kamchatka crab. Biochemistry. 2006; 71:513–519. 16732729 10.1134/s0006297906050075

[226] Anisimova V.E., Rebrikov D.V., Shagin D.A., Kozhemyako V.B., Menzorova N.I., Staroverov D.B., Ziganshin R., Vagner L.L., Rasskazov V.A., Lukyanov S.A. Isolation, characterization and molecular cloning of duplex-specific nuclease from the hepatopancreas of the Kamchatka crab. BMC Biochem. 2008; 9:14. 18495036 10.1186/1471-2091-9-14 PMC2413221

[227] Shagin D.A., Rebrikov D.V., Kozhemyako V.B., Altshuler I.M., Shcheglov A.S., Zhulidov P.A., Bogdanova E.A., Staroverov D.B., Rasskazov V.A., Lukyanov S. A novel method for SNP detection using a new duplex-specific nuclease from crab hepatopancreas. Genome Res. 2002; 12:1935–1942. 12466298 10.1101/gr.547002 PMC187582

[228] Bogdanova E.A., Shagin D.A., Lukyanov S.A. Normalization of full-length enriched cDNA. Mol. Biosyst. 2008; 4:205–212. 18437263 10.1039/b715110c

[229] Bogdanov E.A., Shagina I., Barsova E.V., Kelmanson I., Shagin D.A., Lukyanov S.A. Normalizing cDNA libraries. Curr. Protoc. Mol. Biol. 2010; 5:5.12.1–5.12.27. 10.1002/0471142727.mb0512s90 20373503

[230] Christodoulou D.C., Gorham J.M., Herman D.S., Seidman J.G. Construction of normalized RNA-seq libraries for next-generation sequencing using the crab duplex-specific nuclease. Curr. Protoc. Mol. Biol. 2011; 4, doi:10.1002/0471142727.mb0412s94. 10.1002/0471142727.mb0412s94 PMC3152986 21472699

[231] Shagina I., Bogdanova E., Mamedov I.Z., Lebedev Y., Lukyanov S., Shagin D. Normalization of genomic DNA using duplex-specific nuclease. Biotechniques. 2010; 48:455–459. 20569220 10.2144/000113422

[232] Matvienko M., Kozik A., Froenicke L., Lavelle D., Martineau B., Perroud B., Michelmore R. Consequences of normalizing transcriptomic and genomic libraries of plant genomes using a duplex-specific nuclease and tetramethylammonium chloride. PLoS One. 2013; 8:e55913. 23409088 10.1371/journal.pone.0055913 PMC3568094

[233] Melchior W.B., Von Hippel P.H. Alteration of the relative stability of dA-dT and dG-dC base pairs in DNA. Proc. Natl. Acad. Sci. U.S.A. 1973; 70:298–302. 4346879 10.1073/pnas.70.2.298 PMC433243

[234] Wood W.I., Gitschier J., Lasky L.A., Lawn R.M. Base composition-independent hybridization in tetramethylammonium chloride: a method for oligonucleotide screening of highly complex gene libraries. Proc. Natl. Acad. Sci. U.S.A. 1985; 82:1585–1588. 3856838 10.1073/pnas.82.6.1585 PMC397316

[235] Honoré B., Madsen P., Leffers H. The tetramethylammonium chloride method for screening of cDNA libraries using highly degenerate oligonucleotides obtained by backtranslation of amino-acid sequences. J. Biochem. Biophys. Methods. 1993; 27:39–48. 8409209 10.1016/0165-022x(93)90066-w

[236] Chevet E., Lemaître G., Katinka M.D. Low concentrations of tetramethylammonium chloride increase yield and specificity of PCR. Nucleic Acids Res. 1995; 23:3343–3344. 7667112 10.1093/nar/23.16.3343 PMC307197

[237] Faircloth B.C., Glenn T.C. Not all sequence tags are created equal: designing and validating sequence identification tags robust to indels. PLoS One. 2012; 7:e42543. 22900027 10.1371/journal.pone.0042543 PMC3416851

[238] Islam S., Kjällquist U., Moliner A., Zajac P., Fan J.-B., Lönnerberg P., Linnarsson S. Characterization of the single-cell transcriptional landscape by highly multiplex RNA-seq. Genome Res. 2011; 21:1160–1167. 21543516 10.1101/gr.110882.110 PMC3129258

[239] Shishkin A.A., Giannoukos G., Kucukural A., Ciulla D., Busby M., Surka C., Chen J., Bhattacharyya R.P., Rudy R.F., Patel M.M. Simultaneous generation of many RNA-seq libraries in a single reaction. Nat. Methods. 2015; 12:323–325. 25730492 10.1038/nmeth.3313 PMC4712044

[240] Narayan A., Bommakanti A., Patel A.A. High-throughput RNA profiling via up-front sample parallelization. Nat. Methods. 2015; 12:343–346. 25730493 10.1038/nmeth.3311 PMC4451056

[241] Van Nieuwerburgh F., Soetaert S., Podshivalova K., Ay-Lin Wang E., Schaffer L., Deforce D., Salomon D.R., Head S.R., Ordoukhanian P. Quantitative bias in Illumina TruSeq and a novel post amplification barcoding strategy for multiplexed DNA and small RNA deep sequencing. PLoS One. 2011; 6:e26969. 22046424 10.1371/journal.pone.0026969 PMC3203936

[242] Casbon J.A., Osborne R.J., Brenner S., Lichtenstein C.P. A method for counting PCR template molecules with application to next-generation sequencing. Nucleic Acids Res. 2011; 39:e81. 21490082 10.1093/nar/gkr217 PMC3130290

[243] Jabara C.B., Jones C.D., Roach J., Anderson J.A., Swanstrom R. Accurate sampling and deep sequencing of the HIV-1 protease gene using a Primer ID. Proc. Natl. Acad. Sci. U.S.A. 2011; 108:20166–20171. 22135472 10.1073/pnas.1110064108 PMC3250168

[244] Kivioja T., Vähärautio A., Karlsson K., Bonke M., Enge M., Linnarsson S., Taipale J. Counting absolute numbers of molecules using unique molecular identifiers. Nat. Methods. 2011; 9:72–74. 22101854 10.1038/nmeth.1778

[245] Shiroguchi K., Jia T.Z., Sims P.A., Xie X.S. Digital RNA sequencing minimizes sequence-dependent bias and amplification noise with optimized single-molecule barcodes. Proc. Natl. Acad. Sci. U.S.A. 2012; 109:1347–1352. 22232676 10.1073/pnas.1118018109 PMC3268301

[246] Lundberg D.S., Yourstone S., Mieczkowski P., Jones C.D., Dangl J.L. Practical innovations for high-throughput amplicon sequencing. Nat. Methods. 2013; 10:999–1002. 23995388 10.1038/nmeth.2634

[247] Best K., Oakes T., Heather J.M., Shawe-Taylor J., Chain B. Computational analysis of stochastic heterogeneity in PCR amplification efficiency revealed by single molecule barcoding. Sci. Rep. 2015; 5:14629. 26459131 10.1038/srep14629 PMC4602216

[248] Kinde I., Wu J., Papadopoulos N., Kinzler K.W., Vogelstein B. Detection and quantification of rare mutations with massively parallel sequencing. Proc. Natl. Acad. Sci. U.S.A. 2011; 108:9530–9535. 21586637 10.1073/pnas.1105422108 PMC3111315

[249] Shugay M., Britanova O.V., Merzlyak E.M., Turchaninova M.A., Mamedov I.Z., Tuganbaev T.R., Bolotin D.A., Staroverov D.B., Putintseva E.V., Plevova K. Towards error-free profiling of immune repertoires. Nat. Methods. 2014; 11:653–655. 24793455 10.1038/nmeth.2960

[250] Turchaninova M.A., Davydov A., Britanova O.V., Shugay M., Bikos V., Egorov E.S., Kirgizova V.I., Merzlyak E.M., Staroverov D.B., Bolotin D.A. High-quality full-length immunoglobulin profiling with unique molecular barcoding. Nat. Protoc. 2016; 11:1599–1616. 27490633 10.1038/nprot.2016.093

[251] Deakin C.T., Deakin J.J., Ginn S.L., Young P., Humphreys D., Suter C.M., Alexander I.E., Hallwirth C.V. Impact of next-generation sequencing error on analysis of barcoded plasmid libraries of known complexity and sequence. Nucleic Acids Res. 2014; 42:e129. 25013183 10.1093/nar/gku607 PMC4176369

[252] Brodin J., Hedskog C., Heddini A., Benard E., Neher R.A., Mild M., Albert J. Challenges with using primer IDs to improve accuracy of next generation sequencing. PLoS One. 2015; 10:e0119123. 25741706 10.1371/journal.pone.0119123 PMC4351057

[253] Glanville J., D’Angelo S., Khan T.A., Reddy S.T., Naranjo L., Ferrara F., Bradbury A. Deep sequencing in library selection projects: what insight does it bring. Curr. Opin. Struct. Biol. 2015; 33:146–160. 26451649 10.1016/j.sbi.2015.09.001 PMC4648538

[254] Krueger F., Andrews S.R., Osborne C.S. Large scale loss of data in low-diversity illumina sequencing libraries can be recovered by deferred cluster calling. PLoS One. 2011; 6:e16607. 21305042 10.1371/journal.pone.0016607 PMC3030592

[255] Cornman R.S., Otto C.R.V., Iwanowicz D., Pettis J.S. Taxonomic characterization of honey bee (Apis mellifera) pollen foraging based on non-overlapping paired-end sequencing of nuclear ribosomal loci. PLoS One. 2015; 10:e0145365. 26700168 10.1371/journal.pone.0145365 PMC4689544

[256] Boyle P., Clement K., Gu H., Smith Z.D., Ziller M., Fostel J.L., Holmes L., Meldrim J., Kelley F., Gnirke A. Gel-free multiplexed reduced representation bisulfite sequencing for large-scale DNA methylation profiling. Genome Biol. 2012; 13:R92. 23034176 10.1186/gb-2012-13-10-r92 PMC3491420

[257] Faith J.J., Guruge J.L., Charbonneau M., Subramanian S., Seedorf H., Goodman A.L., Clemente J.C., Knight R., Heath A.C., Leibel R.L. The long-term stability of the human gut microbiota. Science. 2013; 341:1237439. 23828941 10.1126/science.1237439 PMC3791589

[258] Wu L., Wen C., Qin Y., Yin H., Tu Q., Van Nostrand J.D., Yuan T., Yuan M., Deng Y., Zhou J. Phasing amplicon sequencing on Illumina Miseq for robust environmental microbial community analysis. BMC Microbiol. 2015; 15:125. 26084274 10.1186/s12866-015-0450-4 PMC4472414

